# Synthesis and biological evaluation of *N*-cyanoalkyl-, *N*-aminoalkyl-, and *N*-guanidinoalkyl-substituted 4-aminoquinoline derivatives as potent, selective, brain permeable antitrypanosomal agents

**DOI:** 10.1016/j.bmc.2016.08.036

**Published:** 2016-11-01

**Authors:** Irene Sola, Albert Artigas, Martin C. Taylor, F. Javier Pérez-Areales, Elisabet Viayna, M. Victòria Clos, Belén Pérez, Colin W. Wright, John M. Kelly, Diego Muñoz-Torrero

**Affiliations:** a Laboratory of Pharmaceutical Chemistry (CSIC Associated Unit), Faculty of Pharmacy and Food Sciences, and Institute of Biomedicine (IBUB), University of Barcelona, Av. Joan XXIII, 27-31, E-08028 Barcelona, Spain; b Department of Pathogen Molecular Biology, London School of Hygiene and Tropical Medicine, Keppel Street, London WC1E 7HT, United Kingdom; c Department of Pharmacology, Therapeutics and Toxicology, Institute of Neurosciences, Autonomous University of Barcelona, E-08193, Bellaterra, Barcelona, Spain; d Bradford School of Pharmacy, University of Bradford, West Yorkshire BD7 1 DP, United Kingdom

**Keywords:** 4-Aminoquinolines, Side chain modification, Guanidines, Antitrypanosomal agents, Brain permeability

## Abstract

Current drugs against human African trypanosomiasis (HAT) suffer from several serious drawbacks. The search for novel, effective, brain permeable, safe, and inexpensive antitrypanosomal compounds is therefore an urgent need. We have recently reported that the 4-aminoquinoline derivative huprine Y, developed in our group as an anticholinesterasic agent, exhibits a submicromolar potency against *Trypanosoma brucei* and that its homo- and hetero-dimerization can result in to up to three-fold increased potency and selectivity. As an alternative strategy towards more potent smaller molecule anti-HAT agents, we have explored the introduction of ω-cyanoalkyl, ω-aminoalkyl, or ω-guanidinoalkyl chains at the primary amino group of huprine or the simplified 4-aminoquinoline analogue tacrine. Here, we describe the evaluation of a small in-house library and a second generation of newly synthesized derivatives, which has led to the identification of 13 side chain modified 4-aminoquinoline derivatives with submicromolar potencies against *T. brucei*. Among these compounds, the guanidinononyltacrine analogue **15e** exhibits a 5-fold increased antitrypanosomal potency, 10-fold increased selectivity, and 100-fold decreased anticholinesterasic activity relative to the parent huprine Y. Its biological profile, lower molecular weight relative to dimeric compounds, reduced lipophilicity, and ease of synthesis, make it an interesting anti-HAT lead, amenable to further optimization to eliminate its remaining anticholinesterasic activity.

## Graphical abstract

**Figure:**
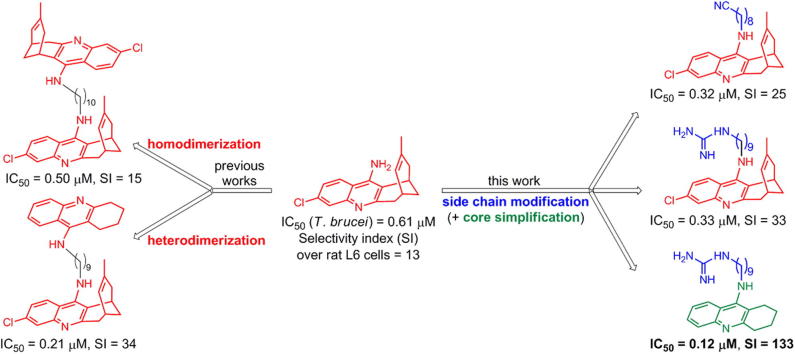

## 1 Introduction

Human African trypanosomiasis (HAT or sleeping sickness) is one of the 17 infectious diseases grouped under the term *Neglected Tropical Diseases*, which inflict a devastating effect on the health and economy of nearly 150 countries.^1^, ^2^, ^3^, ^4^ HAT is caused by two subspecies of the protozoan parasite *Trypanosoma brucei*, which are transmitted to humans through the bite of tsetse flies in rural areas of sub-Saharan Africa. The two subspecies of this parasite lead to distinct disease courses and display different geographical distribution. Most cases of HAT occur in western and central Africa and are due to *T. brucei gambiense*, which causes a chronic infection that slowly progresses from an initial hemolymphatic stage, often asymptomatic, to a late stage, in which the parasites spread into the central nervous system. This produces severe neurological pathology, including sleep disruptions, which give rise to the common name of the disease. About 2–5% of HAT cases occur in southern and eastern Africa and are caused by *T. brucei rhodesiense*. This leads to an acute infection that rapidly progresses from early to late stage disease. With both forms of HAT, the absence of effective treatment in the late stage inexorably leads to coma and death.^5^

Over the last 15 years, because of public health measures, there has been considerable success combatting HAT, with the estimated numbers of those infected falling from 300,000 to less than 20,000.^6^ However, the disease still occurs in 36 countries, with 65 million people at risk, and there is a constant potential for large epidemic outbreaks.

Vaccines are not a realistic option for prevention of HAT because of antigenic variation in the parasite.^7^ Chemotherapy is therefore of particular importance.^8^ Unfortunately, the few drugs that have been approved for HAT (pentamidine and suramin for early stage HAT; melarsoprol and eflornithine, alone or in combination with nifurtimox, for late stage HAT) are unsatisfactory for several reasons, which include the occurrence of major side effects, high costs associated with parenteral administration and medical supervision, lack of brain permeability (in the case of pentamidine and suramin), which precludes their use in late stage HAT, and the increasing emergence of resistance.^9^, ^10^, ^11^ Thus, the development of novel antitrypanosomal compounds that can overcome these issues is urgently needed.^3^, ^12^

Repurposing of known drugs is being increasingly pursued for antitrypanosomal drug discovery, particularly because this strategy should be more rapid and less expensive than the development of new chemical entities.^4^, ^13^, ^14^, ^15^ Despite this, most research efforts to replenish the antitrypanosomal pipeline remain focussed on the development of novel compounds, rationally designed or screened against one or several parasite biological targets^16^, ^17^, ^18^, ^19^ or, more often, arising from phenotypic whole cell screens of compound libraries.^20^, ^21^, ^22^, ^23^, ^24^, ^25^, ^26^

**Figure 1:**
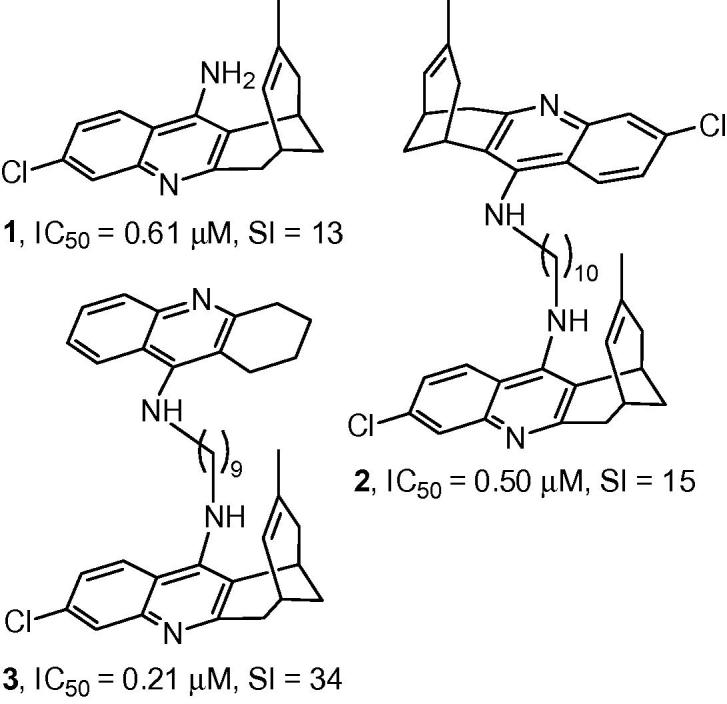
Structures, trypanocidal (*T. brucei*) activities, and selectivity indices of huprine Y, **1**, and the oligomethylene-linked homo- and heterodimers **2** and **3**.

7-Chloro-4-aminoquinoline derivatives are within those structural classes that are being developed for the treatment of HAT.^27^ We recently reported that huprine Y (**1**, Fig. 1), a 7-chloro-4-aminoquinoline derivative with potent acetylcholinesterase (AChE) inhibitory activity, developed in our group as an anti-Alzheimer drug candidate,^28^ exhibited significant activity against *T. brucei* (IC_50_ = 0.61 μM, selectivity index (SI) over rat myoblast L6 cells = 13).^29^, ^30^ In 4-aminoquinoline-based antimalarials, both dimerization and side chain modification have been used to increase potency and overcome parasite resistance.^31^, ^32^, ^33^, ^34^, ^35^ Interestingly, we have found that homodimerization of huprine Y (as in compound **2**,^36^ Fig. 1) and heterodimerization with the 4-aminoquinoline derivative tacrine (as in compound **3**,^37^ Fig. 1) also results in up to 3-fold increased potency and selectivity against *T. brucei*.

**Figure 2:**
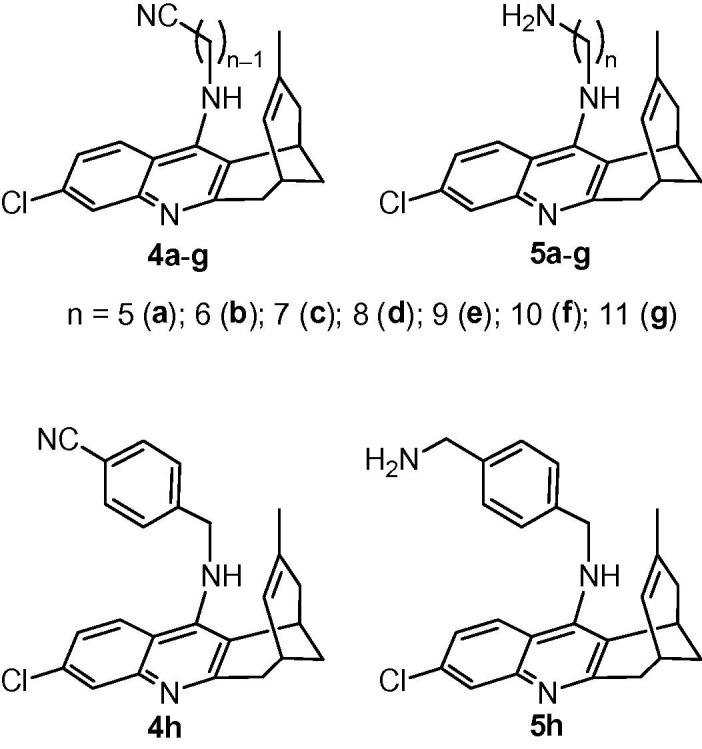
Structures of cyanoalkyl- and aminoalkyl-huprines **4a**–**h** and **5a**–**h**.

Modification of the side chain attached to the exocyclic amino group of 7-chloro-4-aminoquinoline derivatives has also been reported to lead to increased antitrypanosomal activity.^35^ To explore further the structure–antitrypanosomal activity relationships around the huprine scaffold, we report here: (i) the screening of a series of huprine derivatives, substituted at the exocyclic amino group with cyanoalkyl or aminoalkyl chains of different lengths and nature (nitriles **4a**–**h**, and amines **5a**–**h**, Fig. 2), against cultured bloodstream forms of *T. brucei*, rat skeletal myoblast L6 cells, and electric eel AChE, and the evaluation of their brain permeability using an in vitro artificial membrane assay (PAMPA-BBB); (ii) the synthesis and evaluation of the antitrypanosomal, cytotoxic, and anticholinesterasic activity and brain permeability of novel huprine and structurally related tacrine derivatives with other modified side chains terminating in cyano, primary or cyclic amino, or guanidino groups.

## 2 Results and discussion

### 2.1 Screening of the antitrypanosomal, cytotoxic, and anticholinesterasic activity and brain permeability of the ω-cyanoalkyl- and ω-aminoalkyl-huprines **4a**–**h** and **5a**–**h**

The ω-aminoalkyl-huprines **5a**–**h** (Fig. 2) were recently synthesized in our group as immediate precursors of a family of huprine-based anti-Alzheimer hybrid compounds.^38^ We inferred that these compounds might be interesting antitrypanosomal leads based on two grounds. Firstly, aminoalkylhuprines **5a**–**h** should be diprotonated at physiological pH, like pentamidine and other antitrypanosomal dicationic compounds,^39^, ^40^ which seemed favourable for anti-HAT activity. Secondly, we expected that the substitution of one of the lipophilic 4-aminoquinoline moieties of bis(4-aminoquinoline) dimers, like **2** and **3** (Fig. 1), by a primary amino group in aminoalkylhuprines **5a**–**h** would result in a decreased AChE inhibitory activity, and hence, a lower risk of unwanted cholinergic side-effects. Indeed, this trend in AChE inhibitory activity has been reported for a family of dimeric tacrines,^41^ and can be ascribed to a less efficient interaction with a secondary binding site of the enzyme AChE, the so-called peripheral anionic site, when the second 4-aminoquinoline moiety of the dimer, bis(7)tacrine, is substituted by a simple primary amino group.

Because some nitriles have been found to display antitrypanosomal activity,^9^, ^42^ we also envisaged the biological screening of the nitriles **4a**–**h** (Fig. 2), the synthetic precursors of amines **5a**–**h**.^38^

The ω-cyanoalkyl-huprines **4a**–**h** and the ω-aminoalkyl-huprines **5a**–**h** were first screened against the bloodstream form of *T. brucei*, the clinically relevant form of the parasite,^43^ using nifurtimox and huprine Y as reference compounds. These side chain modified huprine derivatives displayed low micromolar to submicromolar IC_50_ values, with all of them being more potent antitrypanosomal agents than nifurtimox, and a few being slightly more potent than, or equipotent to, the parent huprine Y (Table 1). Somewhat unexpectedly, nitriles were found to be in general more potent than the corresponding amines, especially those featuring hepta- to nona-methylene side chains (octa- to deca-methylene side chains in the amines), which were 4–8-fold more potent than their amine counterparts. A clear trend was found in the antitrypanosomal potency of nitriles **4a**–**h** regarding the length of the linker, with the potency increasing from *n* = 5 (**4a**) to *n* = 9 (**4e**), and then decreasing for the longer homologues **4f** and **4g**. For the amines, the highest potency was found for the heptamethylene-linked derivative **5c**. The presence of a *p*-phenylene ring in the side chain does not seem to be of particular relevance for the antitrypanosomal activity, with the *p*-phenylene-linked nitrile **4h** and amine **5h** being equipotent to nitrile **4a** and amine **5a** with a similar side chain length.

**Table 1:** Antitrypanosomal, cytotoxic, and anticholinesterasic activity and BBB permeability of cyanoalkylhuprines **4a**–**h** and aminoalkylhuprines **5a**–**h** and reference compounds **1** and nifurtimox^a^

Compd	*T. brucei* IC_50_ (μM)	*T. brucei* IC_90_ (μM)	L6 cells IC_50_ (μM)	SI*_Tb_*^b^	*Ee*AChE IC_50_ (nM)	*P*_e_ (10^−6^ cm s^−1^)^c^ (prediction)
**4a**	2.13 ± 0.49	4.04 ± 0.06	20.3 ± 0.2	9.7	26.9 ± 1.1	d
**4b**	1.60 ± 0.13	3.21 ± 0.32	19.0 ± 0.3	11.9	31.1 ± 3.1	12.4 ± 0.6 (CNS+)
**4c**	0.86 ± 0.05	1.22 ± 0.03	11.6 ± 3.0	13.5	d	d
**4d**	0.62 ± 0.02	0.85 ± 0.01	7.65 ± 0.41	12.3	d	d
**4e**	0.32 ± 0.01	0.42 ± 0.01	8.04 ± 0.53	25.1	d	d
**4f**	0.46 ± 0.01	0.61 ± 0.01	4.79 ± 0.25	10.4	9.67 ± 0.89	11.3 ± 1.2 (CNS+)
**4g**	1.37 ± 0.06	1.91 ± 0.02	4.98 ± 0.21	3.6	d	16.2 ± 1.3 (CNS+)
**4h**	1.86 ± 0.08	3.39 ± 0.15	10.0 ± 0.8	5.4	158 ± 21	19.3 ± 1.2 (CNS+)
**5a**	0.92 ± 0.08	2.92 ± 0.43	3.82 ± 0.11	4.2	d	d
**5b**	2.03 ± 0.10	3.09 ± 0.21	4.78 ± 0.16	2.4	d	d
**5c**	0.68 ± 0.20	1.15 ± 0.05	4.33 ± 0.08	6.4	36.0 ± 3.5	7.6 ± 0.7 (CNS+)
**5d**	2.28 ± 0.29	4.28 ± 0.46	12.5 ± 3.3	5.5	17.3 ± 1.2	9.8 ± 0.6 (CNS+)
**5e**	2.61 ± 0.16	4.58 ± 0.31	13.6 ± 3.4	5.2	16.4 ± 1.8	7.1 ± 0.7 (CNS+)
**5f**	3.33 ± 0.10	4.33 ± 0.18	7.55 ± 0.19	2.3	20.3 ± 2.5	7.0 ± 0.3 (CNS+)
**5g**	1.79 ± 0.03	2.24 ± 0.04	8.06 ± 0.63	4.5	35.3 ± 3.9	4.0 ± 0.2 (CNS±)
**5h**	0.92 ± 0.03	1.44 ± 0.15	4.27 ± 0.13	4.6	d	11.7 ± 1.1 (CNS+)
**1**^e^	0.61 ± 0.03	2.94 ± 0.20	7.80 ± 0.47	13	0.30 ± 0.01	23.8 ± 2.7 (CNS+)^f^
Nifurtimox	4.4 ± 0.7^g^		32.0 ± 1.1	7.3	d	d

a In vitro activity against bloodstream form of *T. brucei* (pH 7.4), rat myoblast L6 cells, and *Electrophorus electricus* AChE, expressed as the concentration that inhibited growth or enzyme activity by 50% (IC_50_) and 90% (IC_90_, for *T. brucei*). Data are the mean of triplicate experiments ± SEM.

b SI*_Tb_*: selectivity index as the ratio of cytotoxic to anti-*T. brucei* IC_50_ values.

c Permeability values from the PAMPA-BBB assay. Values are expressed as the mean ± SD of three independent experiments.

d Not determined.

e Trypanocidal and cytotoxicity activity values taken from Ref. 29.

f Taken from Ref. 38.

g Taken from Ref. 44.

Thus, the most interesting side chain modified huprine derivative was nitrile **4e**, which, with an IC_50_ value against *T. brucei* of 320 nM (and an IC_90_ value of 420 nM), was 14-fold more potent than nifurtimox and 2-fold more potent than huprine Y (7-fold more potent than huprine Y in terms of the IC_90_ values), in agreement with the expected increase in antitrypanosomal potency upon modification of the side chain at the exocyclic amino group.

Interestingly, nitriles **4a**–**h** and amines **5a**–**h** turned out to be less toxic to rat skeletal myoblast L6 cells than to *T. brucei*, with nitriles **4a**–**h** being less cytotoxic than the corresponding amines **5a**–**h** (Table 1), especially nitrile **4e**, which displayed a selectivity index of 25, i.e. 2- and 3.5-fold more than that of the parent huprine Y and nifurtimox, respectively.

As expected, the introduction of the ω-cyanoalkyl and ω-aminoalkyl chains at the primary amino group of huprine Y led to a clear decrease in AChE inhibitory activity (up to 500-fold). Notwithstanding the lower AChE inhibitory potency relative to the parent huprine Y, nitriles **4a**–**h** and amines **5a**–**h** were more potent AChE inhibitors than would be desirable in antitrypanosomal agents, with nanomolar IC_50_ values for electric eel AChE inhibition (Table 1).

Because good brain penetration is necessary for the treatment of late-stage HAT, the brain permeability of nitriles **4a**–**h** and amines **5a**–**h** was assessed in vitro through the well-established parallel artificial membrane permeability assay (PAMPA-BBB).^45^ As with the parent huprine Y, the permeabilities of most of these modified analogues, through the porcine brain lipid extract used as an artificial blood–brain barrier (BBB) model, were found to be above the threshold established for high BBB permeation (CNS+, *P_e_* (10^−6^ cm s^−1^) > 5.17, Table 1). Therefore, these compounds are predicted to be able to cross the BBB, with nitriles **4a**–**h** being more permeable than the corresponding amines (Table 1), probably due to the dicationic character of the latter at physiological pH.

Overall, the screening of this small in-house compound library pointed to neutral cyano or basic primary amino groups at the end of a side chain of 9 or 7 carbon atoms, respectively, as being favourable substitution patterns for potent, selective, and brain permeable antitrypanosomal agents.

### 2.2 Synthesis of novel side chain modified 4-aminoquinoline derivatives

**Scheme 1:**
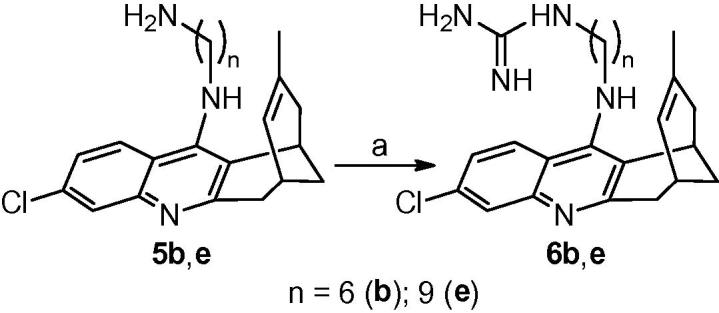
Reagents and conditions: (a) 1*H*-pyrazole-1-carboxamidine hydrochloride, Et_3_N, CH_3_CN, reflux, overnight.

**Scheme 2:**
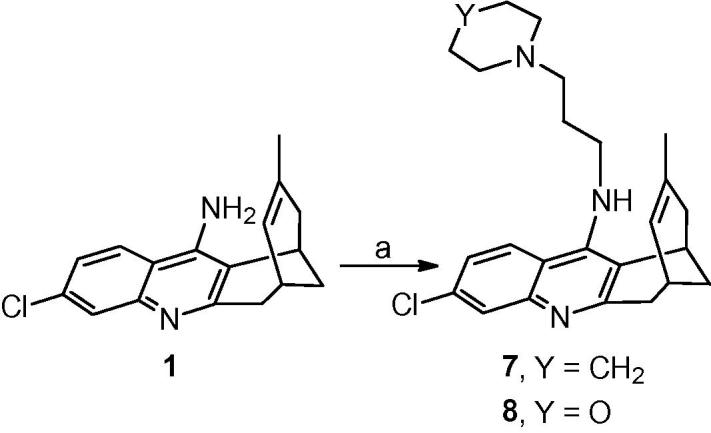
Reagents and conditions: (a) KOH, 4 Å molecular sieves, DMSO, rt, 2 h; then, 1*-*(3-chloropropyl)piperidine hydrochloride or 3-morpholinopropyl methanesulfonate, DMSO, rt, overnight.

Even though the ω-aminoalkyl-huprines **5a**–**h** turned out to be less potent antitrypanosomal agents than the corresponding nitriles **4a**–**h**, they still displayed a submicromolar antitrypanosomal potency in some cases, as well as some selectivity and brain permeability. Indeed, other classes of compounds, such as bis-guanidines and bis-amidines, are diprotonated at physiological pH, like aminoalkylhuprines **5a**–**h**, and exhibit potent antitrypanosomal activity,^40^, ^46^, ^47^, ^48^, ^49^ and brain permeability.^40^ To further extend the SAR around side chain modified huprine derivatives, we undertook the synthesis and biological profiling of novel dibasic huprine derivatives featuring a terminal guanidine (**6b** and **6e**, Scheme 1), piperidine (**7**, Scheme 2) or morpholine (**8**, Scheme 2) moiety, together with different side chain lengths.

**Scheme 3:**
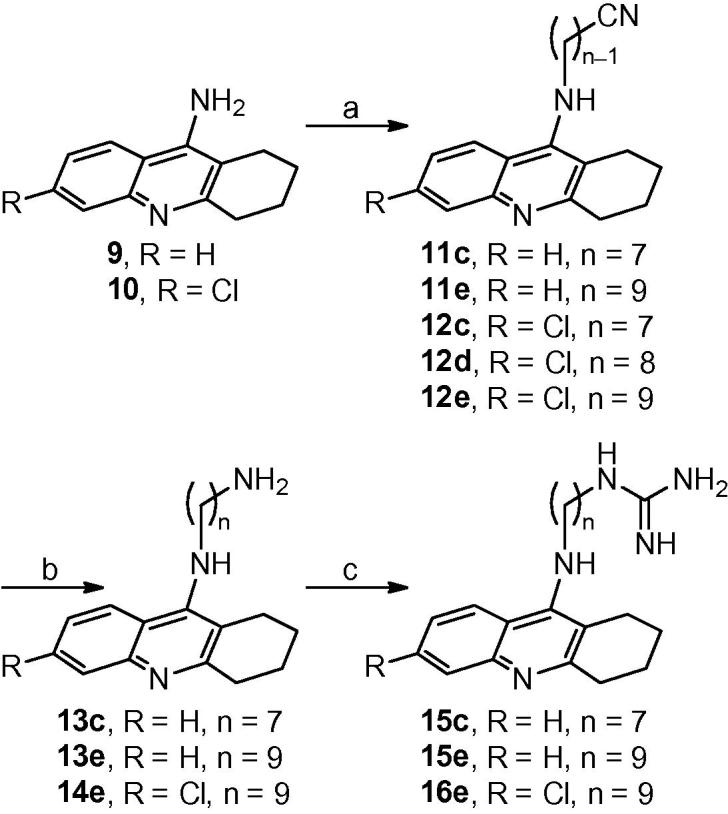
Reagents and conditions: (a) KOH, 4 Å molecular sieves, DMSO, rt, 2 h; then, ω-bromoalkanenitrile, DMSO, rt, overnight; (b) LiAlH_4_, Et_2_O, rt, overnight; (c) 1*H*-pyrazole-1-carboxamidine hydrochloride, Et_3_N, CH_3_CN, reflux, overnight.

In addition, to assess the role of the unsaturated methyl-substituted three-carbon bridge of the huprine moiety, we also synthesised a series of ω-cyanoalkyl-, ω-aminoalkyl-, and ω-guanidinoalkyl derivatives (**11c**, **11e**, **12c**–**e**, **13c**, **13e**, **14e**, **15c**, **15e**, **16e**, Scheme 3), in which the huprine core was substituted by the simpler, less lipophilic (by around 2 log *P* units), and easier-to-synthesize tricyclic core of the 4-aminoquinoline derivatives tacrine and 6-chlorotacrine (**9** and **10**, respectively, Scheme 3). These featured oligomethylene chains of lengths in the range that was found optimal for antitrypanosomal activity in the huprine series (*n* = 7–9).

Guanidinoalkyl huprines **6b** and **6e** were readily synthesized in moderate yield (30% and 59%, respectively) from the corresponding amines **5b** and **5e** upon reaction with 1*H*-pyrazole-1-carboxamidine hydrochloride in dry CH_3_CN in the presence of Et_3_N (Scheme 1).

Likewise, the synthesis of the piperidinopropyl- and morpholinopropyl-huprines **7** and **8** only required one step, i.e. the alkylation of huprine Y, **1**, with the commercial 1*-*(3-chloropropyl)piperidine hydrochloride or the readily available 3-morpholinopropyl methanesulfonate,^50^ after deprotonation of the primary amino group with KOH (Scheme 2).

For the synthesis of the novel cyanoalkyl tacrines **11c**, **11e**, and **12c**–**e**, the known aminoalkyl tacrines **13c**,^41^ **13e**,^51^ and **14e**,^52^ and the novel guanidinoalkyl tacrines **15c**, **15e**, and **16e**, we followed the same three-step protocol that we had used in the huprine series, based on the initial alkylation of tacrine or chlorotacrine with the corresponding ω-bromoalkanenitrile, followed by LiAlH_4_ reduction of the cyano to a primary amino group, and final conversion of the amines into the guanidines upon treatment with 1*H*-pyrazole-1-carboxamidine hydrochloride (Scheme 3).

All the target compounds were transformed into the corresponding hydrochloride or dihydrochloride salts, with which their chemical and biological characterization was performed.

### 2.3 Biological profiling of the novel side chain modified 4-aminoquinoline derivatives

The therapeutic potential of the second generation side chain modified huprine and tacrine derivatives was assessed by evaluation of their antitrypanosomal activity against cultured bloodstream forms of *T. brucei*, and by their brain permeability. Additionally, their potential toxicity was assessed by measuring their effect on the viability of rat L6 cells as a model of normal mammalian cells and by their AChE inhibitory activity.

We found several compounds that exhibited nanomolar antitrypanosomal IC_50_ and IC_90_ values, favourable selectivity indices, and brain permeability (Table 2).

**Table 2:** Antitrypanosomal, cytotoxic, and anticholinesterase activity and BBB permeability of the *N*-cyanoalkyl, *N*-aminoalkyl, and *N*-guanidinoalkyl 4-aminoquinoline derivatives **6b**,**e**, **7**, **8**, **11c**,**e**, **12c**–**e**, **13c**,**e**, **14e**, **15c**,**e**, and **16e** and reference compounds **1** and nifurtimox^a^

Compd	*T. brucei* IC_50_ (μM)	*T. brucei* IC_90_ (μM)	L6 cells IC_50_ (μM)	SI*_Tb_*^b^	*Ee*AChE IC_50_ (nM)	*P*_e_ (10^−6^ cm s^−1^)^c^ (prediction)
**6b**	1.37 ± 0.02	1.54 ± 0.01	21.0 ± 1.6	15.3	11.8 ± 1.3	2.9 ± 0.4 (CNS±)
**6e**	0.33 ± 0.06	0.70 ± 0.09	10.9 ± 0.9	33	10.6 ± 0.8	5.4 ± 0.3 (CNS+)
**7**	0.83 ± 0.04	1.70 ± 0.21	2.40 ± 0.19	2.9	48.4 ± 2.7	5.9 ± 0.4 (CNS+)
**8**	1.75 ± 0.19	4.88 ± 0.27	14.3 ± 0.5	8.2	13.3 ± 0.8	15.0 ± 1.0 (CNS+)
**11c**	3.81 ± 1.13	10.3 ± 2.7	34.8 ± 1.6	9.1	53.9 ± 3.0	11.2 ± 0.2 (CNS+)
**11e**	0.98 ± 0.05	1.38 ± 0.09	5.73 ± 0.16	4.2	44.9 ± 1.6	7.6 ± 0.35 (CNS+)
**12c**	7.92 ± 0.17	12.2 ± 0.2	34.7 ± 0.6	4.4	d	16.7 ± 0.9 (CNS+)
**12d**	6.89 ± 0.13	8.75 ± 0.24	27.8 ± 1.6	4.0	46.0 ± 6.0	14.7 ± 0.8 (CNS+)
**12e**	1.57 ± 0.17	2.56 ± 0.07	8.00 ± 0.44	5.1	24.3 ± 4.5	5.4 ± 0.4 (CNS+)
**13c**	2.13 ± 0.05	3.12 ± 0.14	7.28 ± 0.86	3.4	23.9 ± 2.6	7.4 ± 0.1 (CNS+)
**13e**	4.07 ± 0.12	5.55 ± 0.10	16.6 ± 0.6	4.1	15.6 ± 1.5	6.9 ± 0.5 (CNS+)
**14e**	2.31 ± 0.36	4.88 ± 0.18	7.62 ± 0.81	3.3	14.5 ± 1.6	5.8 ± 0.3 (CNS+)
**15c**	0.85 ± 0.09	1.30 ± 0.20	<1.20	<1.4	23.9 ± 2.6	5.9 ± 0.3 (CNS+)
**15e**	0.12 ± 0.01	0.25 ± 0.04	15.9 ± 0.8	133	30.5 ± 1.9	6.5 ± 0.3 (CNS+)
**16e**	0.63 ± 0.08	0.96 ± 0.02	11.9 ± 1.2	19	16.4 ± 1.5	6.7 ± 0.4 (CNS+)
**1**^e^	0.61 ± 0.03	2.94 ± 0.20	7.80 ± 0.47	13	0.30 ± 0.01	23.8 ± 2.7 (CNS+)^f^
Nifurtimox	4.4 ± 0.7^g^		32.0 ± 1.1	7.3	d	d

a In vitro activity against bloodstream form of *T. brucei* (pH 7.4), rat myoblast L6 cells, and *Electrophorus electricus* AChE, expressed as the concentration that inhibited growth or enzyme activity by 50% (IC_50_) and 90% (IC_90_, for *T. brucei*). Data are the mean of triplicate experiments ± SEM.

b SI*_Tb_*: selectivity index as the ratio of cytotoxic to anti-*T. brucei* IC_50_ values.

c Permeability values from the PAMPA-BBB assay. Values are expressed as the mean ± SD of three independent experiments.

d Not determined.

e Trypanocidal and cytotoxicity activity values taken from Ref. 29.

f Taken from Ref. 38.

g Taken from Ref. 44.

With the huprine derivatives, we found that the introduction of a guanidine at the end of the side chain led to increased antitrypanosomal potencies relative to the corresponding primary amine counterparts, and to roughly equipotent activity relative to the nitriles. Thus, the novel guanidine **6e**, with a total of 9 carbon atoms in the side chain, displayed the same antitrypanosomal potency as the most active compound of the initially screened series, nitrile **4e** (IC_50_ 0.3 μM) (Table 2). The introduction of a piperidino or a morpholino substituent at the side chain of the huprine derivatives **7** and **8** did not seem to confer any particular contribution to the antitrypanosomal properties, with these compounds displaying similar activities to several of the aminoalkylhuprines.

The same trend observed with the huprine derivatives in regard of the terminal functionality and length of the side chain was found in the tacrine derivatives. The order of antitrypanosomal potencies was guanidines > nitriles > amines, with higher potencies for the guanidines and nitriles that have a total number of 9 carbon atoms in the side chain. Higher antitrypanosomal potencies were also found for those derivatives with an unsubstituted tacrine moiety. Thus, the nonamethylene-linked guanidine **15e** (IC_50_ 120 nM) was found to be the most potent side chain modified tacrine derivative (Table 2).

With regard to the 4-aminoquinoline core, replacement of the huprine with the less complex tacrine moiety led, in general, to a drop in antitrypanosomal activity, with ω-aminoalkyl- and ω-cyanoalkyl (6-chloro)tacrines being 1–3-fold and 3–9-fold, respectively, less potent than the corresponding ω-aminoalkyl- and ω-cyanoalkyl huprine derivatives. A notable exception was the ω-guanidinononyl tacrine **15e**, which was 3-fold more potent than the ω-guanidinononyl huprine **6e**. Overall this was the most potent side chain modified 4-aminoquinoline derivative.

Interestingly, all the second generation side chain modified huprine and tacrine derivatives could be inferred to be capable of entering the central nervous system (with the sole exception of guanidine **6b**), on the basis of their measured PAMPA-BBB permeabilities. All of them, with the exception of **15c**, also turned out to be less toxic to mammalian L6 cells than to *T. brucei*, with selectivity indices ranging from 3 to 133 (Table 2). Unfortunately, as with the first generation of screened compounds, despite inhibiting AChE 35–180-fold less potently than the parent compound huprine Y, the anticholinesterasic activity of the second generation side chain modified huprine and tacrine derivatives was still not ideal. Their anticholinesterasic activity was 1–2 orders of magnitude higher than their antitrypanosomal potencies, with the guanidinononyl tacrine **15e** being the best balanced compound (with the anticholinesterasic activity only 4-fold greater than that against typanosomes).

## 3 Conclusion

We recently found that both homodimerization and heterodimerization of the 4-aminoquinoline derivative huprine Y results in increased potency against *T. brucei* and improved selectivity over mammalian cells relative to the parent compound,^36^, ^37^ albeit at the expense of increasing lipophilicity and molecular weight. In this current paper, we have explored the effect on antitrypanosomal activity of the introduction of a side chain, featuring a terminal cyano, amino, or guanidino group, at the primary amino group of huprine Y or the simpler structurally related tacrine or 6-chlorotacrine, as an alternative approach to improve the antitrypanosomal profile. We found that the introduction of a guanidino or a cyano group at the end of a chain of nine carbon atoms was the best type of substitution to produce good antitrypanosomal activity in both the huprine and the tacrine series. The presence of the tetracyclic huprine core leads to higher antitrypanosomal potency in the cyanoalkyl derivatives, whereas the opposite trend was found for guanidinoalkyl derivatives, with the guanidinononyltacrine analogue **15e** being the most promising compound of the side chain modified 4-aminoquinoline derivatives. Overall, 13 out of the 31 side chain modified 4-aminoquinoline derivatives displayed submicromolar potencies against cultured bloodstream form *T. brucei* and good brain permeability. The antitrypanosomal potency of these compounds is greater than their toxicity to mammalian cells, but lower than their anticholinesterasic activity, which might result in undesirable cholinergic side effects.

Guanidinononyltacrine **15e** emerges as the most interesting antitrypanosomal lead of this class. It is endowed with potent (IC_50_ = 120 nM) and selective (SI = 133) activity against *T. brucei*, should be brain permeable, and has the least unfavourable antitrypanosomal/anticholinergic activity ratio. This compound has 5-fold increased antitrypanosomal potency, 10-fold increased selectivity index, and 100-fold decreased anticholinesterasic activity compared with the parent huprine Y, together with lower lipophilicity and molecular weight relative to huprine-based homo- and hetero-dimeric compounds. Our findings confirm that introduction of a conveniently functionalized side chain at the primary amino group of 4-aminoquinoline derivatives may be superior to homo- and hetero-dimerization as a strategy to improve the potential anti-HAT therapeutic profile of this structural class.

## 4 Experimental

### 4.1 Chemistry. General methods

Melting points were determined in open capillary tubes with a MFB 595010M Gallenkamp melting point apparatus. 400 MHz ^1^H/100.6 MHz ^13^C NMR spectra were recorded on a Varian Mercury 400 spectrometer at the Centres Científics i Tecnològics of the University of Barcelona (CCiTUB). The chemical shifts are reported in ppm (*δ* scale) relative to solvent signals (CD_3_OD at 3.31 and 49.0 ppm in the ^1^H and ^13^C NMR spectra, respectively), and coupling constants are reported in Hertz (Hz). The *syn* (*anti*) notation of the protons at position 13 of the huprine moiety of compounds **6b**,**e**, **7**, and **8** means that the corresponding proton at position 13 is on the same (different) side of the quinoline moiety with respect to the cyclohexene ring. IR spectra were run on a Perkin-Elmer Spectrum RX I spectrophotometer. Absorption values are expressed as wavenumbers (cm^−1^); only significant absorption bands are given. Column chromatography was performed on silica gel 60 AC.C (35–70 mesh, SDS, ref 2000027). Thin-layer chromatography was performed with aluminium-backed sheets with silica gel 60 F_254_ (Merck, ref. 1.05554), and spots were visualized with UV light and 1% aqueous solution of KMnO_4_. High resolution mass spectra were carried out at the CCiTUB with a LC/MSD TOF Agilent Technologies spectrometer. The analytical samples of all of the compounds that were subjected to pharmacological evaluation were dried at 65 °C/2 Torr for at least 2 days (standard conditions). Nitriles **11c** and **12c** are protected in a patent of our group,^53^ where, however, no NMR spectra data were given. A more consistent chemical characterization of these compounds is included in this section.

#### 4.1.1 1-{6-[(3-Chloro-6,7,10,11-tetrahydro-9-methyl-7,11-methanocycloocta[*b*]quinolin-12-yl)amino]hexyl}guanidine (**6b**)

To a solution of amine **5b** (462 mg, 1.20 mmol) in dry CH_3_CN (5 mL), anhydrous Et_3_N (0.5 mL, 363 mg, 3.59 mmol) and 1*H*-pyrazole-1-carboxamidine hydrochloride (172 mg, 1.17 mmol) were added. The reaction mixture was stirred under reflux overnight. The resulting precipitated solid was taken in CH_2_Cl_2_ (25 mL) and treated with 2 N NaOH (20 mL). The organic phase was washed with H_2_O (3 × 20 mL), dried over anhydrous Na_2_SO_4_, and concentrated under reduced pressure to afford guanidine **6b** (150 mg, 30% yield), as a brownish solid.

A solution of **6b** (150 mg, 0.39 mmol) in CH_2_Cl_2_ (10 mL) was treated with a methanolic solution of HCl (0.75 N, 4.2 mL, 3.15 mmol) and concentrated under reduced pressure. The resulting solid was taken in MeOH (0.25 mL) and precipitated by addition of EtOAc (1.4 mL). The precipitate was washed with pentane (3 × 2 mL) to give, after drying under standard conditions, **6b**·2HCl (37 mg) as a brownish hygroscopic solid; mp 166–168 °C; IR (ATR) *ν* 3500–2500 (max at 3260, 3134, 2929, 2865, ^+^NH, NH, CH st), 1659, 1631, 1582, 1567, 1514 (C=N, Ar–C–C, Ar–C–N st) cm^−1^; ^1^H NMR (400 MHz, CD_3_OD) *δ* 1.42–1.54 (complex signal, 4H, 3-H_2_, 4-H_2_), 1.58 (s, 3H, 9′-CH_3_), superimposed in part 1.62 (tt, *J* = *J*′ = 6.8 Hz, 2H, 2-H_2_), superimposed in part 1.91 (tt, *J* = *J*′ = 7.2 Hz, 2H, 5-H_2_), 1.93 (br d, *J* = 17.2 Hz, 1H, 10′-H*_endo_*), superimposed in part 1.94 (br d, *J* = 12.8 Hz, 1H, 13′-H*_syn_*), 2.09 (dm, *J* = 12.8 Hz, 1H, 13′-H*_anti_*), 2.56 (dd, *J* = 17.2 Hz, *J*′ = 4.4 Hz, 1H, 10′-H*_exo_*), 2.77 (m, 1H, 7′-H), 2.86 (br d, *J* = 17.6 Hz, 1H, 6′-H*_endo_*), 3.19 (t, *J* = 6.8 Hz, 2H, 1-H_2_), superimposed in part 3.20 (dd, *J* = 17.6 Hz, *J*′ = 5.2 Hz, 1H, 6′-H*_exo_*), 3.46 (m, 1H, 11′-H), 4.00 (t, *J* = 7.2 Hz, 2H, 6-H_2_), 4.85 (s, ^+^NH, ^+^NH_2_, NH, NH_2_), 5.59 (br d, *J* = 4.8 Hz, 1H, 8′-H), 7.57 (dd, *J* = 9.2 Hz, *J*′ = 2.0 Hz, 1H, 2′-H), 7.77 (d, *J* = 2.0 Hz, 1H, 4′-H), 8.41 (d, *J* = 9.2 Hz, 1H, 1′-H); ^13^C NMR (100.6 MHz, CD_3_OD) *δ* 23.5 (CH_3_, 9′-CH_3_), 27.26 (CH, C11′), 27.29 (CH_2_), 27.4 (CH_2_) (C3, C4), 27.8 (CH, C7′), 29.3 (CH_2_, C13′), 29.8 (CH_2_, C2), 31.2 (CH_2_, C5), 36.0 (CH_2_, C6′), 36.1 (CH_2_, C10′), 42.4 (CH_2_, C1), 49.5 (CH_2_, C6), 115.6 (C, C12a′), 117.6 (C, C11a′), 119.1 (CH, C4′), 125.1 (CH, C8′), 126.7 (CH, C2′), 129.5 (CH, C1′), 134.6 (C, C9′), 140.2 (C, C3′), 141.0 (C, C4a′), 151.3 (C, C5a′), 156.8 (C, C12′), 158.6 (C, guanidine C=N); HRMS (ESI), calcd for (C_24_H_32_^35^ClN_5_+H^+^) 426.2419, found 426.2414.

#### 4.1.2 1-{9-[(3-Chloro-6,7,10,11-tetrahydro-9-methyl-7,11-methanocycloocta[*b*]quinolin-12-yl)amino]nonyl}guanidine (**6e**)

It was prepared as described for **6b**. From amine **5e** (50 mg, 0.12 mmol) and 1*H*-pyrazole-1-carboxamidine hydrochloride (21 mg, 0.14 mmol), a brownish solid residue (39 mg) was obtained. This crude product was taken in MeOH (2 mL) and eluted through a Dowex™ Marathon™ A OH^–^ anion exchange resin (5 g) using MeOH (500 mL) as the eluent, to provide **6e** (33 mg, 59% yield) as a brownish oil.

**6e**·2HCl: brownish solid; mp 210–211 °C; IR (ATR) *ν* 3500–2500 (max at 3250, 3137, 2923, 2852, ^+^NH, NH, CH st), 1630, 1577, 1513 (C=N, Ar–C–C, Ar–C–N st) cm^−1^; ^1^H NMR (400 MHz, CD_3_OD) *δ* 1.34–1.48 (complex signal, 10H, 3-H_2_, 4-H_2_, 5-H_2_, 6-H_2_, 7-H_2_), superimposed in part 1.56 (m, 2H, 2-H_2_), 1.59 (s, 3H, 9′-CH_3_), 1.86 (tt, *J* = *J*′ = 7.2 Hz, 2H, 8-H_2_), 1.93 (br d, *J* = 17.6 Hz, 1H, 10′-H*_endo_*), superimposed in part 1.94 (dm, *J* = 12.4 Hz, 1H, 13′-H*_syn_*), 2.09 (dm, *J* = 12.4 Hz, 1H, 13′-H*_anti_*), 2.55 (dd, *J* = 17.6 Hz, *J*′ = 4.4 Hz, 1H, 10′-H*_exo_*), 2.77 (m, 1H, 7′-H), 2.86 (br d, *J* = 18.0 Hz, 1H, 6′-H*_endo_*), 3.16 (t, *J* = 7.2 Hz, 2H, 1-H_2_), 3.21 (dd, *J* = 18.0 Hz, *J*′ = 5.6 Hz, 1H, 6′-H*_exo_*), 3.45 (m, 1H, 11′-H), 3.98 (t, *J* = 7.2 Hz, 2H, 9-H_2_), 4.85 (s, ^+^NH, ^+^NH_2_, NH, NH_2_), 5.59 (br d, *J* = 4.4 Hz, 1H, 8′-H), 7.56 (dd, *J* = 9.2 Hz, *J*′ = 2.4 Hz, 1H, 2′-H), 7.77 (d, *J* = 2.4 Hz, 1H, 4′-H), 8.40 (d, *J* = 9.2 Hz, 1H, 1′-H); ^13^C NMR (100.6 MHz, CD_3_OD) *δ* 23.5 (CH_3_, 9′-CH_3_), 27.3 (CH, C11′), 27.7 (CH_2_), 27.8 [(CH, C7′) + CH_2_] (C3, C7), 29.3 (CH_2_, C13′), 29.9 (CH_2_, C2), 30.3 (2CH_2_), 30.6 (CH_2_) (C4, C5, C6), 31.3 (CH_2_, C8), 36.0 (CH_2_, C6′), 36.1 (CH_2_, C10′), 42.5 (CH_2_, C1), 49.6 (CH_2_, C9), 115.7 (C, C12a′), 117.6 (C, C11a′), 119.1 (CH, C4′), 125.1 (CH, C8′), 126.6 (CH, C2′), 129.5 (CH, C1′), 134.5 (C, C9′), 140.3 (C, C3′), 141.0 (C, C4a′), 151.2 (C, C5a′), 156.9 (C, C12′), 158.6 (C, guanidine C=N); HRMS (ESI), calcd for (C_27_H_38_^35^ClN_5_+H^+^) 468.2889, found 468.2903.

#### 4.1.3 3-Chloro-6,7,10,11-tetrahydro-9-methyl-12-[(3-piperidinopropyl)amino]-7,11-methanocycloocta[*b*]quinoline (**7**)

A mixture of huprine Y, **1** (500 g, 1.76 mmol), finely powdered KOH (85% purity, 346 mg, 5.24 mmol), and 4 Å molecular sieves in anhydrous DMSO (4 mL) was stirred, heating every 10 min approximately with a heat gun for 1 h and at rt for an additional hour, and then treated with a solution of 1*-*(3-chloropropyl)piperidine hydrochloride (429 mg, 2.17 mmol) in anhydrous DMSO (2.5 mL). The reaction mixture was stirred at room temperature overnight, diluted with H_2_O (50 mL) and extracted with CH_2_Cl_2_ (3 × 25 mL). The combined organic extracts were washed with H_2_O (3 × 100 mL), dried over anhydrous Na_2_SO_4_, and evaporated at reduced pressure to give a yellowish solid (413 mg), which was purified by column chromatography (40–60 μm silica gel, CH_2_Cl_2_/MeOH/50% aq NH_4_OH mixtures, gradient elution). On elution with CH_2_Cl_2_/MeOH/50% aq NH_4_OH 99:1:0.4, piperidinopropylhuprine **7** (35 mg, 5% yield) was isolated as a yellowish oil; *R_f_* 0.38 (CH_2_Cl_2_/MeOH/50% aq NH_4_OH 9:1:0.1).

A solution of **7** (74 mg, 0.18 mmol) in CH_2_Cl_2_ (3 mL) was filtered through a 0.2 μm NYL filter, treated with HCl/Et_2_O (2.36 N, 0.16 mL, 0.38 mmol), and evaporated under reduced pressure. The resulting solid was washed with pentane (3 × 2 mL) to give, after drying under standard conditions, **7**·2HCl (81 mg) as a pale yellow solid; mp 189–190 °C (dec.); IR (ATR) *ν* 3500–2500 (max at 3370, 3261, 3070, 2932, ^+^NH, NH, CH st), 1630, 1580, 1513 (Ar–C–C, Ar–C–N st) cm^−1^; ^1^H NMR (400 MHz, CD_3_OD) *δ* 1.59 (s, 3H, 9-CH_3_), superimposed 1.86–1.96 [complex signal, 6H, piperidine 3(5)-H_2_, piperidine 4-H_2_], 1.93 (br d, *J* = 17.2 Hz, 1H, 10-H*_endo_*), superimposed in part 1.97 (dm, *J* = 12.4 Hz, 1H, 13-H*_syn_*), 2.10 (dm, *J* = 12.4 Hz, 1H, 13-H*_anti_*), 2.37 (tt, *J* = 7.6 Hz, *J*′ = 7.2 Hz, 2H, 2′-H_2_), 2.59 (dd, *J* = 17.2 Hz, *J*′ = 4.8 Hz, 1H, 10-H*_exo_*), 2.79 (m, 1H, 7-H), 2.89 (br d, *J* = 17.6 Hz, 1H, 6-H*_endo_*), 2.90–3.06 [br signal, 2H, piperidine 2(6)-H*_ax_*], 3.23 (dd, *J* = 17.6 Hz, *J*′ = 5.6 Hz, 1H, 6-H*_exo_*), 3.25 (t, *J* = 7.6 Hz, 2H, 3′-H_2_), 3.53 (m, 1H, 11-H), 3.50–3.62 [br signal, 2H, piperidine 2(6)-H*_eq_*], 4.08 (t, *J* = 7.2 Hz, 2H, 1′-H_2_), 4.85 (s, ^+^NH, NH), 5.59 (br d, *J* = 4.8 Hz, 1H, 8-H), 7.61 (dd, *J* = 9.2 Hz, *J*′ = 2.0 Hz, 1H, 2-H), 7.79 (d, *J* = 2.0 Hz, 1H, 4-H), 8.41 (d, *J* = 9.2 Hz, 1H, 1-H); ^13^C NMR (100.6 MHz, CD_3_OD) *δ* 22.7 (CH_2_, piperidine C4), 23.4 (CH_3_, 9-CH_3_), 24.2 [2CH_2_, piperidine C3(5)], 26.0 (CH_2_, C2′), 27.3 (CH, C11), 27.8 (CH, C7), 29.2 (CH_2_, C13), 36.1 (CH_2_, C6), 36.3 (CH_2_, C10), 46.5 (CH_2_, C1′), 54.4 [2CH_2_, piperidine C2(6)], 55.3 (CH_2_, C3′), 115.8 (C, C12a), 118.3 (C, C11a), 119.2 (CH, C4), 125.1 (CH, C8), 127.1 (CH, C2), 129.2 (CH, C1), 134.6 (C, C9), 140.3 (C, C3), 140.9 (C, C4a), 151.8 (C, C5a), 156.8 (C, C12); HRMS (ESI), calcd for (C_25_H_32_^35^ClN_3_+H^+^) 410.2358, found 410.2365.

#### 4.1.4 (±)-3-Chloro-6,7,10,11-tetrahydro-9-methyl-12-[(3-morpholinopropyl)amino]-7,11-methanocycloocta[*b*]quinoline (**8**)

It was prepared as described for **7**. From **1** (500 mg, 1.76 mmol) and 3-morpholinopropyl methanesulfonate (470 mg, 2.10 mmol), a brownish oily residue (543 mg) was obtained and purified by column chromatography (40–60 μm silica gel, CH_2_Cl_2_/50% aq NH_4_OH 100:0.4), to give morpholinopropylhuprine **8** (121 mg, 17% yield) as a yellow oil; *R_f_* 0.42 (CH_2_Cl_2_/MeOH/50% aq NH_4_OH 9:1:0.1).

**8**·2HCl: pale yellow solid; mp 173–174 °C (dec.); IR (ATR) *ν* 3500–2500 (max at 3384, 3225, 3109, 3048, 2922, 2790, 2728, 2683, 2610, ^+^NH, NH, CH st), 1631, 1582, 1563, 1512 (Ar–C–C, Ar–C–N st) cm^−1^; ^1^H NMR (400 MHz, CD_3_OD) *δ* 1.59 (s, 3H), 1.93 (br d, *J* = 17.2 Hz, 1H, 10-H*_endo_*), superimposed in part 1.97 (dm, *J* = 12.8 Hz, 1H, 13-H*_syn_*), 2.10 (dm, *J* = 12.8 Hz, 1H, 13-H*_anti_*), 2.39 (tt, *J* = *J*′ = 7.2 Hz, 2H, 2′-H_2_), 2.59 (dd, *J* = 17.2 Hz, *J*′ = 4.8 Hz, 1H, 10-H*_exo_*), 2.78 (m, 1H, 7-H), 2.89 (br d, *J* = 18.0 Hz, 1H, 6-H*_endo_*), superimposed 3.12–3.22 (m, 2H, 3′-H_2_), 3.23 (dd, *J* = 18.0 Hz, *J*′ = 5.2 Hz, 1H, 6-H*_exo_*), superimposed with the CD_3_OD signal 3.28–3.36 [br signal, 2H, morpholine 3(5)-H*_ax_*], 3.53 (m, 1H, 11-H), superimposed 3.46–3.58 (br signal, 2H, morpholine 3(5)-H*_eq_*], 3.82–3.93 [br signal, 2H, morpholine 2(6)-H*_ax_*], 4.02–4.08 [br signal, 2H, morpholine 2(6)-H*_eq_*], 4.10 (t, *J* = 7.2 Hz, 2H, 1′-H_2_), 4.85 (s, ^+^NH, NH), 5.59 (br d, *J* = 4.8 Hz, 1H, 8-H), 7.61 (dd, *J* = 9.6 Hz, *J*′ = 2.0 Hz, 1H, 2-H), 7.79 (d, *J* = 2.0 Hz, 1H, 4-H), 8.41 (d, *J* = 9.6 Hz, 1H, 1-H); ^13^C NMR (100.6 MHz, CD_3_OD) *δ* 23.5 (CH_3_, 9-CH_3_), 25.7 (CH_2_, C2′), 27.3 (CH, C11), 27.9 (CH, C7), 29.2 (CH_2_, C13), 36.1 (CH_2_, C6), 36.4 (CH_2_, C10), 46.5 (CH_2_, C1′), 53.3 [2CH_2_, morpholine C3(5)], 55.6 (CH_2_, C3′), 65.1 [2CH_2_, morpholine C2(6)], 115.8 (C, C12a), 118.3 (C, C11a), 119.3 (CH, C4), 125.1 (CH, C8), 127.2 (CH, C2), 129.3 (CH, C1), 134.7 (C, C9), 140.3 (C, C3), 140.9 (C, C4a), 151.8 (C, C5a), 156.9 (C, C12); HRMS (ESI), calcd for (C_24_H_30_^35^ClN_3_O+H^+^) 412.2150, found 412.2164.

#### 4.1.5 7-[(1,2,3,4-Tetrahydroacridin-9-yl)amino]heptanenitrile (**11c**)^53^

A suspension of tacrine, **9** (1.70 g, 8.57 mmol) and finely powdered KOH (85% purity, 0.97 g, 14.7 mmol), and 4 Å molecular sieves in anhydrous DMSO (20 mL) was stirred, heating every 10 min approximately with a heat gun for 1 h and at room temperature one additional hour, and then treated with a solution of 7-bromoheptanenitrile (90% purity, 1.55 mL, 1.96 g, 9.28 mmol) in anhydrous DMSO (12 mL) dropwise during 30 min. The reaction mixture was stirred at room temperature overnight, diluted with 5 N NaOH (50 mL) and extracted with EtOAc (3 × 150 mL). The combined organic extracts were washed with H_2_O (4 × 100 mL), dried over anhydrous Na_2_SO_4_, and evaporated under reduced pressure to give a crude product (2.53 g). Purification of this residue by column chromatography (40–60 μm silica gel, CH_2_Cl_2_/50% aq NH_4_OH 100:0.2) afforded a 85:15 mixture of the dialkylated byproduct and nitrile **11c** (190 mg) and pure nitrile **11c** (1.75 g, 66% isolated yield) as a yellow oil; *R_f_* 0.77 (CH_2_Cl_2_/MeOH/50% aq NH_4_OH 9:1:0.05).

A solution of **11c** (79 mg, 0.26 mmol) in CH_2_Cl_2_ (15 mL) was filtered through a 0.2 μm NYL filter, treated with methanolic HCl (0.53 N, 2.15 mL, 1.14 mmol) and evaporated under reduced pressure. The resulting solid was taken in MeOH (0.20 mL) and precipitated by addition of EtOAc (0.60 mL). The precipitate was washed with pentane (3 × 2 mL) to give, after drying under standard conditions, **11c**·HCl (39 mg) as a pale brown sticky solid; IR (ATR) *ν* 3500–2500 (max at 3237, 2932, 2860, 2770, ^+^NH, NH, CH st), 2242 (CN st), 1633, 1586, 1573, 1523 (Ar–C–C, Ar–C–N st) cm^−1^; ^1^H NMR (400 MHz, CD_3_OD) *δ* 1.46–1.54 (complex signal, 4H, 4-H_2_, 5-H_2_), 1.66 (tt, *J* = 7.2 Hz, *J*′ = 6.8 Hz, 2H, 3-H_2_), 1.86 (tt, *J* = 7.2 Hz, *J*′ = 6.8 Hz, 2H, 6-H_2_), 1.94–2.02 (complex signal, 4H, 2′-H_2_, 3′-H_2_), 2.44 (t, *J* = 6.8 Hz, 2H, 2-H_2_), 2.71 (br t, *J* = 5.6 Hz, 2H, 1′-H_2_), 3.02 (br t, *J* = 6.0 Hz, 2H, 4′-H_2_), 3.97 (t, *J* = 7.2 Hz, 2H, 7-H_2_), 4.85 (s, ^+^NH, NH), 7.59 (dd, *J* = 8.4 Hz, *J*′ = 7.6 Hz, 1H, 7′-H), 7.77 (d, *J* = 8.8 Hz, 1H, 5′-H), 7.85 (dd, *J* = 8.8 Hz, *J*′ = 7.6 Hz, 1H, 6′-H), 8.40 (d, *J* = 8.4 Hz, 1H, 8′-H); ^13^C NMR (100.6 MHz, CD_3_OD) *δ* 17.2 (CH_2_, C2), 21.8 (CH_2_, C3′), 23.0 (CH_2_, C2′), 24.9 (CH_2_, C1′), 26.3 (CH_2_, C3), 26.9 (CH_2_, C5), 29.30 (CH_2_), 29.33 (CH_2_) (C4, C4′), 31.2 (CH_2_, C6), 49.0 (CH_2_, C7), 112.9 (C, C9a′), 117.1 (C, C8a′), 120.1 (CH, C5′), 121.1 (C, C1), 126.3 (CH, C7′), 126.5 (CH, C8′), 134.1 (CH, C6′), 139.8 (C, C10a′), 151.7 (C, C4a′), 158.0 (C, C9′); HRMS (ESI), calcd for (C_20_H_25_N_3_+H^+^) 308.2121, found 308.2117.

#### 4.1.6 9-[(1,2,3,4-Tetrahydroacridin-9-yl)amino]nonanenitrile (**11e**)

It was prepared as described for **11c**. From **9** (500 mg, 2.52 mmol) and 9-bromononanenitrile (659 mg, 3.02 mmol), a yellowish oily residue (984 mg) was obtained and purified by column chromatography (40–60 μm silica gel, CH_2_Cl_2_/MeOH/50% aq NH_4_OH mixtures, gradient elution). On elution with CH_2_Cl_2_/MeOH/50% aq NH_4_OH 99.9:0.1:0.4 to 99.7:0.3:0.4, nitrile **11e** (272 mg, 32% yield) was isolated as a yellow oil; *R_f_* 0.51 (CH_2_Cl_2_/MeOH/50% aq NH_4_OH 9:1:0.1).

**11e**·HCl: yellow sticky solid; IR (ATR) *ν* 3500–2500 (max at 3268, 2931, 2848, 2756, 2700, 2667, ^+^NH, NH, CH st), 2241 (CN st), 1630, 1590, 1572, 1522 (Ar–C–C, Ar–C–N st) cm^−1^; ^1^H NMR (400 MHz, CD_3_OD) *δ* 1.34–1.48 (complex signal, 8H, 4-H_2_, 5-H_2_, 6-H_2_, 7-H_2_), 1.62 (tt, *J* = *J*′ = 7.2 Hz, 2H, 3-H_2_), 1.84 (tt, *J* = *J*′ = 7.2 Hz, 2H, 8-H_2_), 1.94–2.00 (complex signal, 4H, 2′-H_2_, 3′-H_2_), 2.42 (t, *J* = 7.2 Hz, 2H, 2-H_2_), 2.71 (br t, *J* = 5.6 Hz, 2H, 1′-H_2_), 3.02 (br t, *J* = 6.0 Hz, 2H, 4′-H_2_), 3.96 (t, *J* = 7.2 Hz, 2H, 9-H_2_), 4.85 (s, ^+^NH, NH), 7.59 (ddd, *J* = 8.4 Hz, *J*′ = 7.2 Hz, *J*″ = 1.2 Hz, 1H, 7′-H), 7.75 (br d, *J* = 7.6 Hz, 1H, 5′-H), 7.85 (ddd, *J* = 7.6 Hz, *J*′ = 7.2 Hz, *J*″ = 1.2 Hz, 1H, 6′-H), 8.40 (br d, *J* = 8.4 Hz, 1H, 8′-H); ^13^C NMR (100.6 MHz, CD_3_OD) *δ* 17.3 (CH_2_, C2), 21.8 (CH_2_, C3′), 23.0 (CH_2_, C2′), 24.8 (CH_2_, C1′), 26.3 (CH_2_, C3), 27.5 (CH_2_, C7), 29.3 (CH_2_, C4′), 29.5 (CH_2_), 29.6 (CH_2_), 29.9 (CH_2_) (C4, C5, C6), 31.4 (CH_2_, C8), 49.1 (CH_2_, C9), 112.8 (C, C9a′), 117.0 (C, C8a′), 120.1 (CH, C5′), 121.3 (C, C1), 126.3 (CH, C7′), 126.5 (CH, C8′), 134.1 (CH, C6′), 139.8 (C, C10a′), 151.7 (C, C4a′), 158.0 (C, C9′); HRMS (ESI), calcd for (C_22_H_29_N_3_+H^+^) 336.2434, found 336.2436.

#### 4.1.7 7-[(6-Chloro-1,2,3,4-tetrahydroacridin-9-yl)amino]heptanenitrile (**12c**)^53^

It was prepared as described for **11c**. From 6-chlorotacrine, **10** (2.00 g, 8.59 mmol), and 7-bromoheptanenitrile (90% purity, 1.55 mL, 1.96 g, 9.28 mmol), a yellow oily residue (3.06 g) was obtained. Purification of this residue by column chromatography (40–60 μm silica gel, CH_2_Cl_2_/50% aq NH_4_OH 100:0.2) afforded a 87:13 mixture of dialkylated byproduct and nitrile **12c** (789 mg) and pure nitrile **12c** (2.06 g, 70% isolated yield) as a yellow oil; *R_f_* 0.92 (CH_2_Cl_2_/MeOH/50% aq NH_4_OH 9:1:0.05).

**12c**·HCl: yellowish solid; mp 86–87 °C; IR (ATR) *ν* 3500–2500 (max at 3347, 3138, 3059, 2949, 2928, 2858, 2744, ^+^NH, NH, CH st), 2245 (CN st), 1639, 1605, 1573, 1524 (Ar–C–C, Ar–C–N st) cm^−1^; ^1^H NMR (400 MHz, CD_3_OD) *δ* 1.46–1.54 (complex signal, 4H, 4-H_2_, 5-H_2_), 1.66 (tt, *J* = 7.2 Hz, *J*′ = 6.8 Hz, 2H, 3-H_2_), 1.87 (tt, *J* = *J*′ = 7.2 Hz, 2H, 6-H_2_), 1.92–2.02 (complex signal, 4H, 2′-H_2_, 3′-H_2_), 2.45 (t, *J* = 7.2 Hz, 2H, 2-H_2_), 2.69 (br t, *J* = 6.0 Hz, 2H, 1′-H_2_), 3.01 (br t, *J* = 5.6 Hz, 2H, 4′-H_2_), 3.96 (t, *J* = 7.2 Hz, 2H, 7-H_2_), 4.84 (s, ^+^NH, NH), 7.56 (dd, *J* = 9.2 Hz, *J*′ = 2.4 Hz, 1H, 7′-H), 7.79 (d, *J* = 2.4 Hz, 1H, 5′-H), 8.39 (d, *J* = 9.2 Hz, 1H, 8′-H); ^13^C NMR (100.6 MHz, CD_3_OD) *δ* 17.2 (CH_2_, C2), 21.7 (CH_2_, C3′), 22.9 (CH_2_, C2′), 24.8 (CH_2_, C1′), 26.3 (CH_2_, C3), 26.9 (CH_2_, C5), 29.29 (CH_2_), 29.34 (CH_2_) (C4, C4′), 31.1 (CH_2_, C6), 49.1 (CH_2_, C7), 113.4 (C), 115.4 (C) (C8a′, C9a′), 119.1 (CH, C5′), 121.2 (C, C1), 126.8 (CH, C7′), 128.8 (CH, C8′), 140.1 (CH, C6′), 140.5 (C, C10a′), 152.2 (C, C4a′), 157.8 (C, C9′); HRMS (ESI), calcd for (C_20_H_24_^35^ClN_3_+H^+^) 342.1732, found 342.1737.

#### 4.1.8 8-[(6-Chloro-1,2,3,4-tetrahydroacridin-9-yl)amino]octanenitrile (**12d**)

It was prepared as described for **11c**. From 6-chlorotacrine, **10** (1.00 g, 4.30 mmol), and 8-bromooctanenitrile (1.01 g, 4.95 mmol), a yellow oily residue (1.73 g) was obtained. Purification of this crude by column chromatography (40–60 μm silica gel, CH_2_Cl_2_/50% aq NH_4_OH 100:0.4), afforded nitrile **12d** (411 mg, 27% yield) as a yellow oil; *R_f_* 0.80 (CH_2_Cl_2_/MeOH/50% aq NH_4_OH 9:1:0.1).

**12d**·HCl: yellowish solid; mp 210–213 °C; IR (ATR) *ν* 3500–2500 (max at 3251, 3052, 2934, 2853, 2711, ^+^NH, NH, CH st), 2246 (CN st), 1633, 1616, 1588, 1567, 1542, 1517 (Ar–C–C, Ar–C–N st) cm^−1^; ^1^H NMR (400 MHz, CD_3_OD) *δ* 1.40–1.52 (complex signal, 6H, 4-H_2_, 5-H_2_, 6-H_2_), 1.63 (tt, *J* = *J*′ = 7.2 Hz, 2H, 3-H_2_), 1.85 (tt, *J* = *J*′ = 7.2 Hz, 2H, 7-H_2_), 1.92–2.00 (complex signal, 4H, 2′-H_2_, 3′-H_2_), 2.43 (t, *J* = 7.2 Hz, 2H, 2-H_2_), 2.68 (br t, *J* = 6.0 Hz, 2H, 1′-H_2_), 3.00 (br t, *J* = 6.0 Hz, 2H, 4′-H_2_), 3.95 (t, *J* = 7.2 Hz, 2H, 8-H_2_), 4.85 (s, ^+^NH, NH), 7.57 (dd, *J* = 9.2 Hz, *J*′ = 2.0 Hz, 1H, 7′-H), 7.77 (d, *J* = 2.0 Hz, 1H, 5′-H), 8.39 (d, *J* = 9.2 Hz, 1H, 8′-H); ^13^C NMR (100.6 MHz, CD_3_OD) *δ* 17.2 (CH_2_, C2), 21.8 (CH_2_, C3′), 22.9 (CH_2_, C2′), 24.7 (CH_2_, C1′), 26.3 (CH_2_, C3), 27.4 (CH_2_, C6), 29.36 (CH_2_), 29.40 (CH_2_) (C4, C4′), 29.5 (CH_2_, C5), 31.2 (CH_2_, C7), 49.2 (CH_2_, C8), 113.4 (C), 115.5 (C) (C8a′, C9a′), 119.2 (CH, C5′), 121.2 (C, C1), 126.8 (CH, C7′), 128.8 (CH, C8′), 140.1 (CH, C6′), 140.6 (C, C10a′), 152.2 (C, C4a′), 157.8 (C, C9′); HRMS (ESI), calcd for (C_21_H_26_^35^ClN_3_+H^+^) 356.1888, found 356.1878.

#### 4.1.9 9-[(6-Chloro-1,2,3,4-tetrahydroacridin-9-yl)amino]nonanenitrile (**12e**)

It was prepared as described for **11c**. From 6-chlorotacrine, **10** (500 mg, 2.15 mmol), and 9-bromononanenitrile (561 mg, 2.57 mmol), a yellow oily residue (849 mg) was obtained and purified by column chromatography (40–60 μm silica gel, CH_2_Cl_2_/MeOH/50% aq NH_4_OH mixtures, gradient elution). On elution with CH_2_Cl_2_/MeOH/50% aq NH_4_OH 100:0:0.4 to 99.8:0.2:0.4, nitrile **12e** (380 mg, 48% yield) was isolated as a yellow oil; *R_f_* 0.82 (CH_2_Cl_2_/MeOH/50% aq NH_4_OH 9:1:0.1).

**12e**·HCl: yellowish solid; mp 176–177 °C; IR (ATR) *ν* 3500–2500 (max at 3248, 3048, 2931, 2852, 2714, ^+^NH, NH, CH st), 2246 (CN st), 1632, 1589, 1566, 1523 (Ar–C–C, Ar–C–N st) cm^−1^; ^1^H NMR (400 MHz, CD_3_OD) *δ* 1.36–1.50 (complex signal, 8H, 4-H_2_, 5-H_2_, 6-H_2_, 7-H_2_), 1.62 (tt, *J* = *J*′ = 7.2 Hz, 2H, 3-H_2_), 1.84 (tt, *J* = *J*′ = 7.2 Hz, 2H, 8-H_2_), 1.92–2.02 (complex signal, 4H, 2′-H_2_, 3′-H_2_), 2.43 (t, *J* = 7.2 Hz, 2H, 2-H_2_), 2.68 (br t, *J* = 6.0 Hz, 2H, 1′-H_2_), 3.00 (br t, *J* = 6.4 Hz, 2H, 4′-H_2_), 3.95 (t, *J* = 7.2 Hz, 2H, 9-H_2_), 4.85 (s, ^+^NH, NH), 7.56 (dd, *J* = 9.2 Hz, *J*′ = 2.0 Hz, 1H, 7′-H), 7.78 (d, *J* = 2.0 Hz, 1H, 5′-H), 8.39 (d, *J* = 9.2 Hz, 1H, 8′-H); ^13^C NMR (100.6 MHz, CD_3_OD) *δ* 17.3 (CH_2_, C2), 21.8 (CH_2_, C3′), 22.9 (CH_2_, C2′), 24.7 (CH_2_, C1′), 26.4 (CH_2_, C3), 27.6 (CH_2_, C7), 29.3 (CH_2_, C4′), 29.6 (CH_2_), 29.7 (CH_2_), 30.0 (CH_2_) (C4, C5, C6), 31.3 (CH_2_, C8), 49.2 (CH_2_, C9), 113.4 (C), 115.5 (C) (C8a′, C9a′), 119.2 (CH, C5′), 121.2 (C, C1), 126.8 (CH, C7′), 128.8 (CH, C8′), 140.1 (CH, C6′), 140.5 (C, C10a′), 152.1 (C, C4a′), 157.9 (C, C9’); HRMS (ESI), calcd for (C_22_H_28_^35^ClN_3_+H^+^) 370.2045, found 370.2037.

#### 4.1.10 1-{7-[(1,2,3,4-Tetrahydroacridin-9-yl)amino]heptyl}guanidine (**15c**)

It was prepared as described for **6b**. From amine **13c** (100 mg, 0.32 mmol) and 1*H*-pyrazole-1-carboxamidine hydrochloride (57 mg, 0.39 mmol), a brownish solid residue (87 mg) was obtained. This crude product was taken in MeOH (2 mL) and eluted through a Dowex™ Marathon™ A OH^–^ anion exchange resin (5 g) using MeOH (500 mL) as the eluent, to provide guanidine **15c** (81 mg, 72% yield) as a brownish oil.

**15c**·2HCl: brownish sticky solid; IR (ATR) *ν* 3500–2500 (max at 3257, 3132, 2930, 2858, ^+^NH, NH, CH st), 1631, 1574, 1520 (C=N, Ar–C–C, Ar–C–N st) cm^−1^; ^1^H NMR (400 MHz, CD_3_OD) *δ* 1.38–1.50 (complex signal, 6H, 3-H_2_, 4-H_2_, 5-H_2_), 1.59 (tt, *J* = *J*′ = 6.8 Hz, 2H, 2-H_2_), 1.86 (tt, *J* = *J*′ = 7.2 Hz, 2H, 6-H_2_), 1.92–2.02 (complex signal, 4H, 2′-H_2_, 3′-H_2_), 2.71 (br t, *J* = 5.2 Hz, 2H, 1′-H_2_), 3.02 (t, *J* = 5.6 Hz, 2H, 4′-H_2_), 3.16 (t, *J* = 6.8 Hz, 2H, 1-H_2_), 3.96 (t, *J* = 7.2 Hz, 2H, 7-H_2_), 4.85 (s, ^+^NH, ^+^NH_2_, NH, NH_2_), 7.59 (ddd, *J* = 8.4 Hz, *J*′ = 6.8 Hz, *J*″ = 1.6 Hz, 1H, 7′-H), 7.76 (dd, *J* = 8.4 Hz, *J*′ = 1.6 Hz, 1H, 5′-H), 7.86 (ddd, *J* = 8.4 Hz, *J*′ = 6.8 Hz, *J*″ = 1.2 Hz, 1H, 6′-H), 8.40 (br d, *J* = 8.4 Hz, 1H, 8′-H); ^13^C NMR (100.6 MHz, CD_3_OD) *δ* 21.8 (CH_2_, C3′), 23.0 (CH_2_, C2′), 24.9 (CH_2_, C1′), 27.57 (CH_2_), 27.63 (CH_2_) (C3, C5), 29.3 (CH_2_, C4′), 29.8 (CH_2_), 29.9 (CH_2_) (C2, C4), 31.5 (CH_2_, C6), 42.4 (CH_2_, C1), 49.1 (CH_2_, C7), 112.8 (C, C9a′), 117.0 (C, C8a′), 120.1 (CH, C5′), 126.3 (CH, C7′), 126.5 (CH, C8′), 134.1 (CH, C6′), 139.8 (C, C10a′), 151.7 (C, C4a′), 158.0 (C, C9′), 158.5 (C, guanidine C=N); HRMS (ESI), calcd for (C_21_H_31_N_5_+H^+^) 354.2652, found 354.2665.

#### 4.1.11 1-{9-[(1,2,3,4-Tetrahydroacridin-9-yl)amino]nonyl}guanidine (**15e**)

It was prepared as described for **6b**. From **13e**·HCl (47 mg, 0.12 mmol) and 1*H*-pyrazole-1-carboxamidine hydrochloride (21 mg, 0.14 mmol), a brownish solid residue (46 mg) was obtained. This crude product was taken in MeOH (2 mL) and eluted through a Dowex™ Marathon™ A OH^–^ anion exchange resin (5 g) using MeOH (500 mL) as the eluent, to provide guanidine **15e** (35 mg, 76% yield) as a brownish oil.

**15e**·2HCl: brownish sticky solid; IR (ATR) *ν* 3500–2500 (max at 3256, 3132, 2926, 2852, ^+^NH, NH, CH st), 1661, 1631, 1575, 1521 (C=N, Ar–C–C, Ar–C–N st) cm^−1^; ^1^H NMR (400 MHz, CD_3_OD) *δ* 1.34–1.48 (complex signal, 10H, 3-H_2_, 4-H_2_, 5-H_2_, 6-H_2_, 7-H_2_), 1.58 (tt, *J* = *J*′ = 6.8 Hz, 2H, 2-H_2_), 1.84 (tt, *J* = *J*′ = 7.2 Hz, 2H, 8-H_2_), 1.94–2.00 (complex signal, 4H, 2′-H_2_, 3′-H_2_), 2.70 (br t, *J* = 6.0 Hz, 2H, 1′-H_2_), 3.02 (br t, *J* = 6.4 Hz, 2H, 4′-H_2_), 3.16 (t, *J* = 6.8 Hz, 2H, 1-H_2_), 3.95 (t, *J* = 7.2 Hz, 2H, 9-H_2_), 4.85 (s, ^+^NH, ^+^NH_2_, NH, NH_2_), 7.59 (ddd, *J* = 8.8 Hz, *J*′ = 6.8 Hz, *J*″ = 1.2 Hz, 1H, 7′-H), 7.76 (dd, *J* = 8.4 Hz, *J*′ = 1.2 Hz, 1H, 5′-H), 7.85 (ddd, *J* = 8.4 Hz, *J*′ = 6.8 Hz, *J*″ = 1.2 Hz, 1H, 6′-H), 8.40 (br d, *J* = 8.8 Hz, 1H, 8′-H); ^13^C NMR (100.6 MHz, CD_3_OD) *δ* 21.8 (CH_2_, C3′), 22.9 (CH_2_, C2′), 24.9 (CH_2_, C1′), 27.6 (2CH_2_, C3, C7), 29.3 (CH_2_, C4′), 29.8 (CH_2_, C2), 30.1 (2CH_2_), 30.4 (CH_2_) (C4, C5, C6), 31.5 (CH_2_, C8), 42.4 (CH_2_, C1), 49.1 (CH_2_, C9), 112.8 (C, C9a′), 116.9 (C, C8a′), 120.1 (CH, C5′), 126.3 (CH, C7′), 126.4 (CH, C8′), 134.1 (CH, C6′), 139.7 (C, C10a′), 151.6 (C, C4a′), 157.9 (C, C9′), 158.5 (C, guanidine C=N); HRMS (ESI), calcd for (C_23_H_35_N_5_+H^+^) 382.2965, found 382.2975.

#### 4.1.12 1-{9-[(6-Chloro-1,2,3,4-tetrahydroacridin-9-yl)amino]nonyl}guanidine (**16e**)

It was prepared as described for **6b**. From **14e**·HCl (90 mg, 0.22 mmol) and 1*H*-pyrazole-1-carboxamidine hydrochloride (39 mg, 0.27 mmol), a brownish solid residue (81 mg) was obtained. This crude product was taken in MeOH (2 mL) and eluted through a Dowex™ Marathon™ A OH^−^ anion exchange resin (5 g) using MeOH (500 mL) as the eluent, to provide guanidine **16e** (61 mg, 67% yield) as a brownish oil.

**16e**·2HCl: brownish solid; mp 209–210 °C; IR (ATR) *ν* 3500–2500 (max at 3253, 3126, 2928, 2855, ^+^NH, NH, CH st), 1631, 1572, 1514 (C=N, Ar–C–C, Ar–C–N st) cm^−1^; ^1^H NMR (400 MHz, CD_3_OD) *δ* 1.38–1.52 (complex signal, 10H, 3-H_2_, 4-H_2_, 5-H_2_, 6-H_2_, 7-H_2_), 1.60 (tt, *J* = *J*′ = 6.8 Hz, 2H, 2-H_2_), 1.86 (tt, *J* = *J*′ = 7.2 Hz, 2H, 8-H_2_), 1.92–2.02 (complex signal, 4H, 2′-H_2_, 3′-H_2_), 2.68 (br t, *J* = 6.0 Hz, 2H, 1′-H_2_), 3.00 (br t, *J* = 6.0 Hz, 2H, 4′-H_2_), 3.17 (t, *J* = 6.8 Hz, 2H, 1-H_2_), 3.95 (t, *J* = 7.2 Hz, 2H, 9-H_2_), 4.85 (s, ^+^NH, ^+^NH_2_, NH, NH_2_), 7.57 (dd, *J* = 9.2 Hz, *J*′ = 2.4 Hz, 1H, 7′-H), 7.79 (d, *J* = 2.4 Hz, 1H, 5′-H), 8.40 (d, *J* = 9.2 Hz, 1H, 8′-H); ^13^C NMR (100.6 MHz, CD_3_OD) *δ* 21.8 (CH_2_, C3′), 22.9 (CH_2_, C2′), 24.8 (CH_2_, C1′), 27.5 (CH_2_), 27.6 (CH_2_) (C3, C7), 29.3 (CH_2_, C4′), 29.7 (2CH_2_), 29.8 (2CH_2_) (C2, C4, C5, C6), 31.3 (CH_2_, C8), 42.4 (CH_2_, C1), 49.2 (CH_2_, C9), 113.3 (C), 115.4 (C) (C8a′, C9a′), 119.1 (CH, C5′), 126.8 (CH, C7′), 128.8 (CH, C8′), 140.0 (CH, C6′), 140.4 (C, C10a′), 152.1 (C, C4a′), 157.7 (C, C9′), 158.6 (C, guanidine C=N); HRMS (ESI), calcd for (C_23_H_34_^35^ClN_5_+H^+^) 416.2576, found 416.2589.

### 4.2 Biological assays

#### 4.2.1 *T. brucei* culturing and evaluation of antitrypanosomal activity

Bloodstream form *T. brucei* (strain 221) was cultured at 37 °C in modified Iscove’s medium.^54^ Trypanocidal activity was assessed by growing parasites in the presence of various concentrations of the novel compounds and determining the levels which inhibited growth by 50% (IC_50_) and 90% (IC_90_). *T. brucei* in the logarithmic phase of growth were diluted back to 2.5 × 10^4^ mL^−1^ and aliquoted into 96-well plates. The compounds were then added at a range of concentrations and the plates incubated at 37 °C. Each drug concentration was tested in triplicate. Resazurin was added after 48 h and the plates incubated for a further 16 h and the plates then read in a Spectramax plate reader. Results were analysed using GraphPad Prism.

#### 4.2.2 Cytotoxic activity against rat skeletal myoblast L6 cells

Cytotoxicity against mammalian cells was assessed using microtitre plates following a described procedure.^55^ Briefly, rat skeletal muscle L6 cells were seeded at 1 × 10^4^ mL^−1^ in 200 μL of growth medium containing different compound concentrations. The plates were incubated for 6 days at 37 °C and 20 μL resazurin was then added to each well. After a further 8 h incubation, the fluorescence was determined using a Spectramax plate reader.

#### 4.2.3 Acetylcholinesterase inhibitory activity

The inhibitory activity against *Electrophorus electricus* (Ee) AChE (Sigma–Aldrich) was evaluated spectrophotometrically by the method of Ellman et al.^56^ The reactions took place in a final volume of 300 μL of 0.1 M phosphate-buffered solution pH 8.0, containing EeAChE (0.03 U/mL) and 333 μM 5,5′-dithiobis(2-nitrobenzoic) acid (DTNB; Sigma–Aldrich) solution used to produce the yellow anion of 5-thio-2-nitrobenzoic acid. Inhibition curves were performed in duplicates using at least 10 increasing concentrations of inhibitors and preincubated for 20 min at 37 °C before adding the substrate. One duplicate sample without inhibitor was always present to yield 100% of AChE activity. Then substrate, acetylthiocholine iodide (450 μM; Sigma–Aldrich), was added and the reaction was developed for 5 min at 37 °C. The colour production was measured at 414 nm using a labsystems Multiskan spectrophotometer.

Data from concentration−inhibition experiments of the inhibitors were calculated by non-linear regression analysis, using the GraphPad Prism program package (GraphPad Software; San Diego, USA), which gave estimates of the IC_50_ (concentration of drug producing 50% of enzyme activity inhibition). Results are expressed as mean ± SEM of at least 4 experiments performed in duplicate.

#### 4.2.4 Determination of brain permeability: PAMPA-BBB assay

The in vitro permeability (*P*_e_) of the novel compounds and fourteen commercial drugs through lipid extract of porcine brain membrane was determined by using a parallel artificial membrane permeation assay.^45^ Commercial drugs and the target compounds were tested using a mixture of PBS/EtOH 70:30. Assay validation was made by comparing experimental and described permeability values of the commercial drugs (Table 3), which showed a good correlation: *P*_e_ (exp) = 1.6079 *P*_e_ (lit) − 1.2585 (*R*^2^ = 0.9217). From this equation and the limits established by Di et al. for BBB permeation, three ranges of permeability were established: compounds of high BBB permeation (CNS+): *P*_e_ (10^−6^ cm s^−1^) > 5.17; compounds of low BBB permeation (CNS−): *P*_e_ (10^−6^ cm s^−1^) < 1.95; and compounds of uncertain BBB permeation (CNS±): 5.17 > *P*_e_ (10^−6^ cm s^−1^) > 1.95.

**Table 3:** Reported and experimental permeabilities (*P*_e_ 10^−6^ cm s^−1^) of 14 commercial drugs used for the PAMPA-BBB assay validation

Compd	Literature value^a^	Experimental value^b^
Cimetidine	0.0	0.7 ± 0.1
Lomefloxacin	1.1	0.7 ± 0.1
Norfloxazin	0.1	0.9 ± 0.1
Ofloxazin	0.8	1.0 ± 0.1
Hydrocortisone	1.9	1.4 ± 0.1
Piroxicam	2.5	2.2 ± 0.1
Clonidine	5.3	6.5 ± 0.1
Corticosterone	5.1	6.7 ± 0.1
Imipramine	13.0	12.3 ± 0.1
Promazine	8.8	13.8 ± 0.3
Progesterone	9.3	16.8 ± 0.3
Desipramine	12.0	17.8 ± 0.1
Testosterone	17.0	26.5 ± 0.4
Verapamil	16.0	28.4 ± 0.5

a Taken from Ref. 45.

b Values are expressed as the mean ± SD of three independent experiments.

## Supplementary data

Supplementary data Spectral data.
