# Supplementary material for: Synthesis and biological evaluation of N-cyanoalkyl-, N-aminoalkyl-, and N-guanidinoalkyl-substituted 4-aminoquinoline derivatives as potent, selective, brain permeable antitrypanosomal agents
Source: Bioorg Med Chem. 2016 Nov 1;24(21):5162–71. doi: 10.1016/j.bmc.2016.08.036 (PMC5080452; doi:10.1016/j.bmc.2016.08.036)
Supplement: Supplementary data — Spectral data. [file mmc1.pdf]

Supplementary Material

Synthesis and biological evaluation of *N*-cyanoalkyl-, *N*-aminoalkyl-, and *N*-guanidinoalkyl-substituted 4-aminoquinoline derivatives as potent, selective, brain permeable antitrypanosomal agents

Irene Sola[a], Albert Artigas[a], Martin C. Taylor[b], F. Javier Pérez-Areales[a], Elisabet Viayna[a], M. Victòria Clos[c], Belén Pérez[c], Colin W. Wright[d], John M. Kelly[b], Diego Muñoz-Torrero[a,*]

[a] *Laboratory of Pharmaceutical Chemistry (CSIC Associated Unit), Faculty of Pharmacy and Food Sciences, and Institute of Biomedicine (IBUB), University of Barcelona, Av. Joan XXIII, 27-31, E-08028, Barcelona, Spain*
[b] *Department of Pathogen Molecular Biology, London School of Hygiene and Tropical Medicine, Keppel Street, London WC1E 7HT, United Kingdom*
[c] *Department of Pharmacology, Therapeutics and Toxicology, Institute of Neurosciences, Autonomous University of Barcelona, E-08193, Bellaterra, Barcelona, Spain*
[d] *Bradford School of Pharmacy, University of Bradford, West Yorkshire BD7 1 DP, United Kingdom*

* Corresponding author. Tel.: +34 934024533; fax: +34 934035941.
E-mail address: dmunoztorrero@ub.edu (D. Muñoz-Torrero).

Copies of ^{1}H and ^{13}C NMR spectra of the new compounds

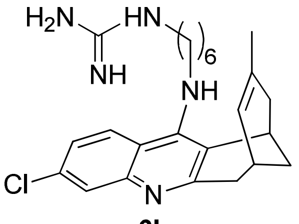

6b

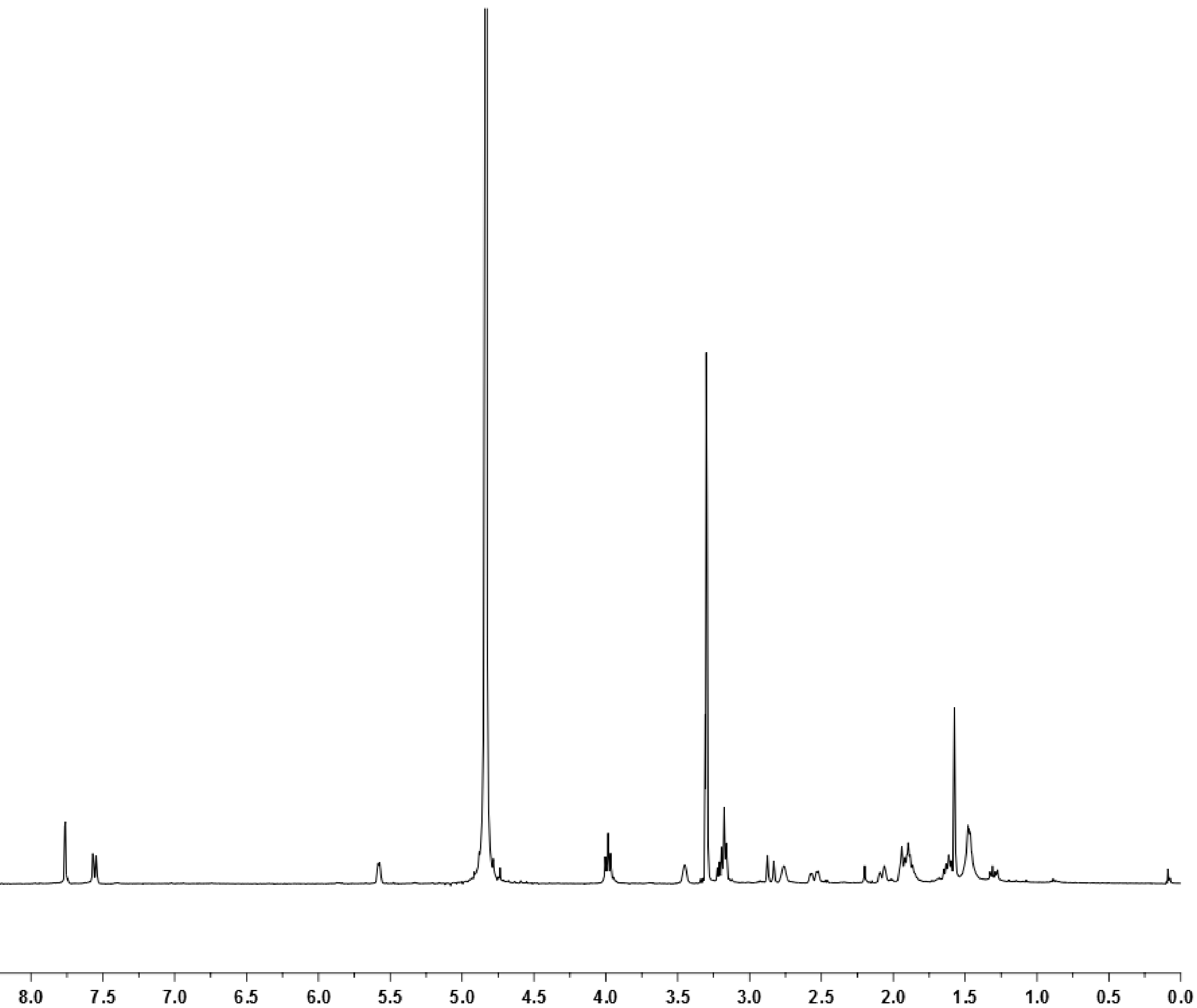

^{1}H NMR (400 MHz, CD_3OD)

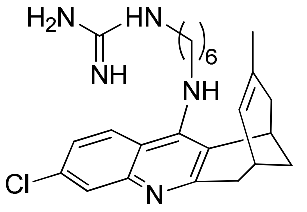

6b

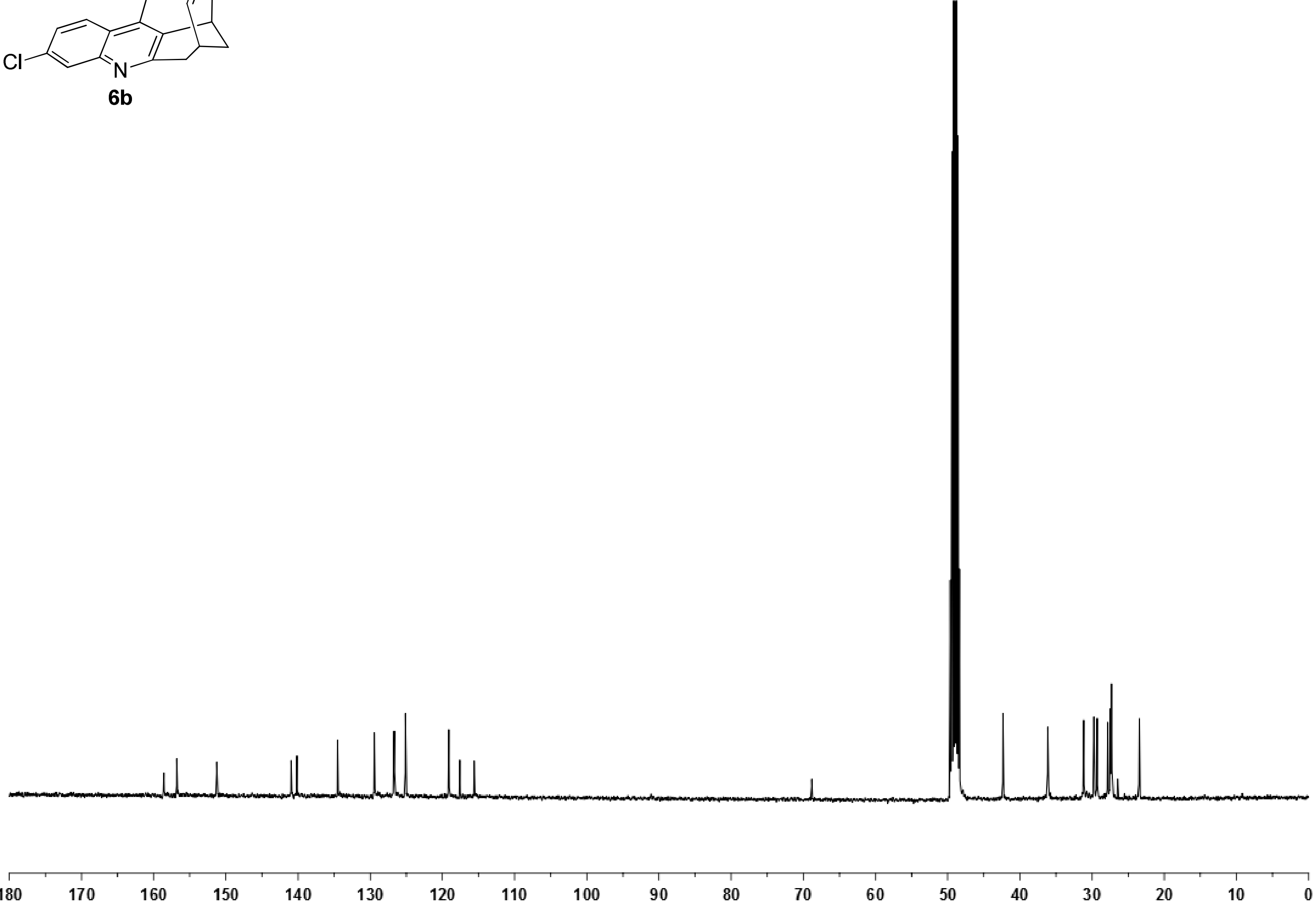

^{13}C NMR (100.6 MHz, CD_3OD)

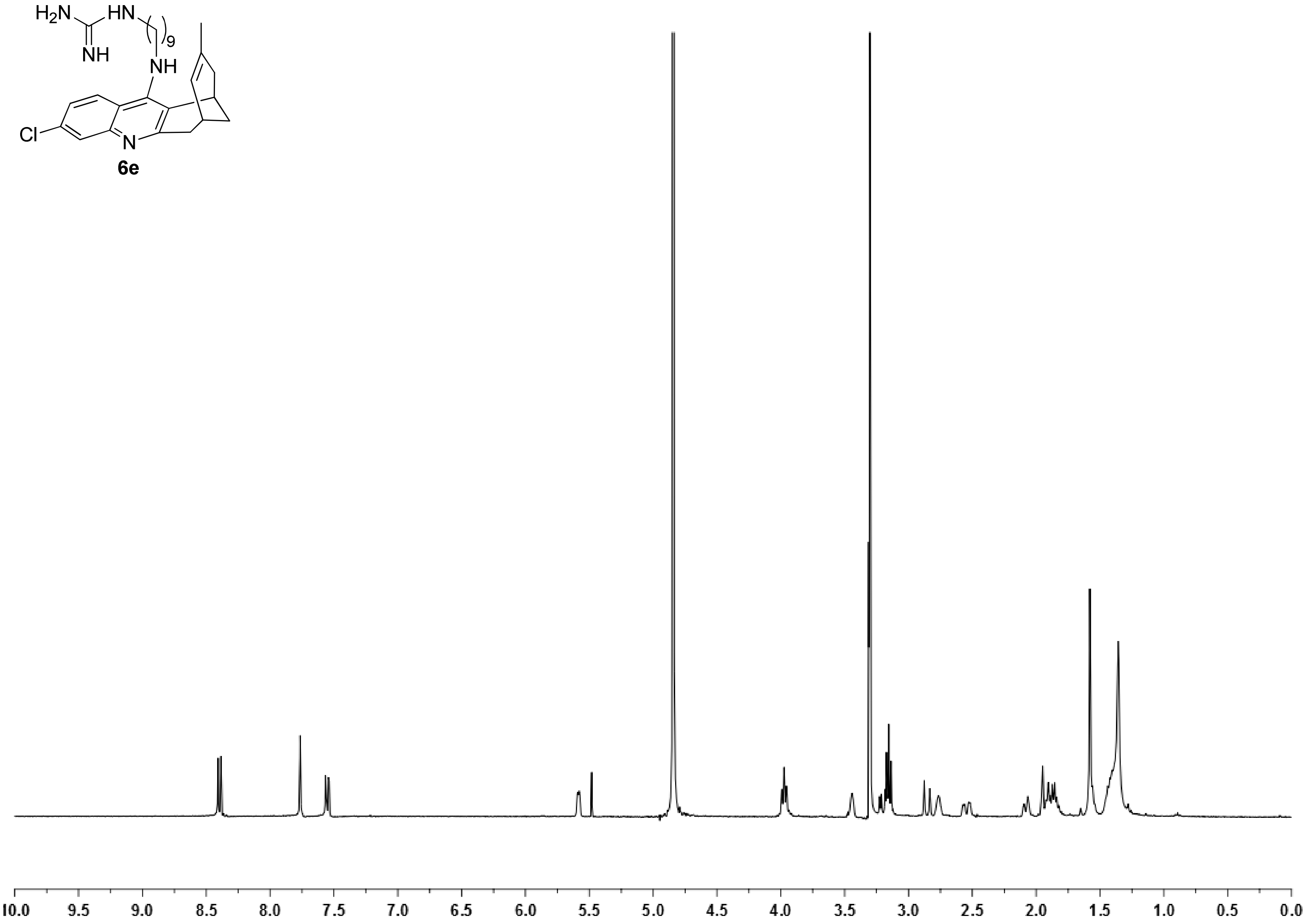

^1H NMR (400 MHz, CD_3OD)

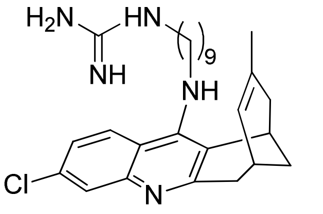

6e

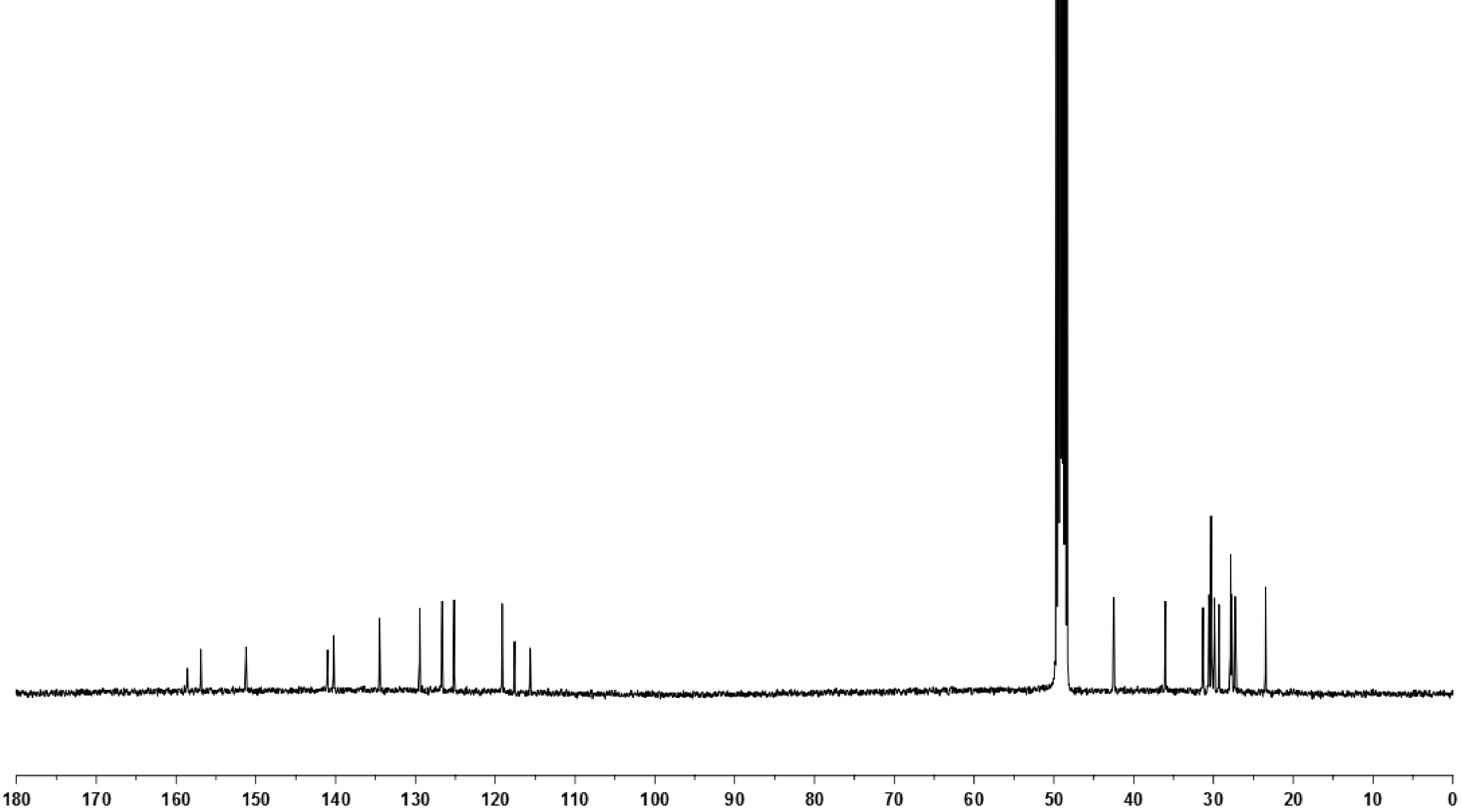

^{13}C NMR (100.6 MHz, CD_3OD)

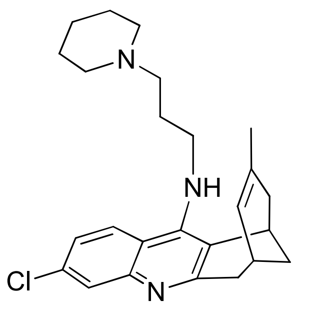

7

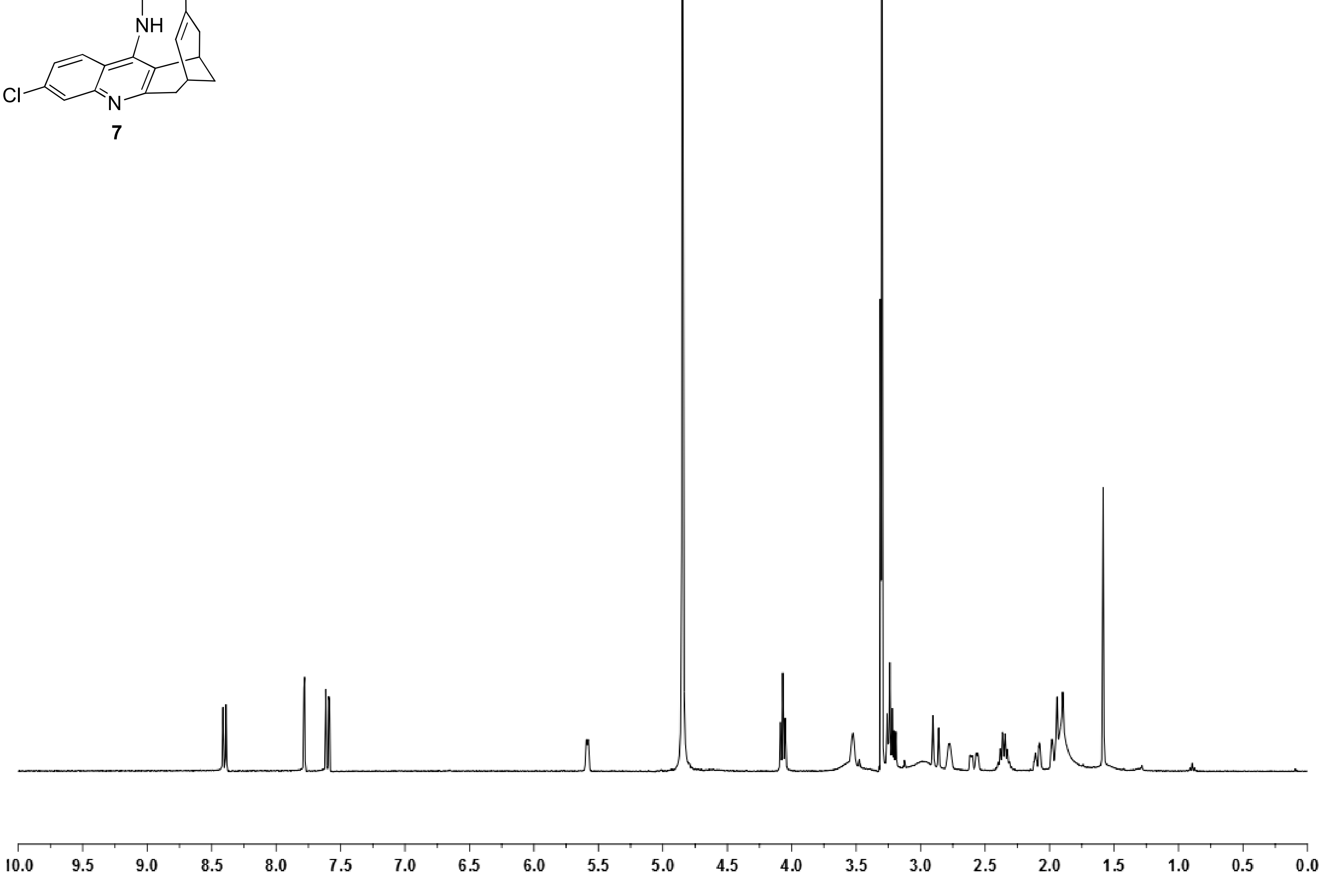

^{1}H NMR (400 MHz, CD_3OD)

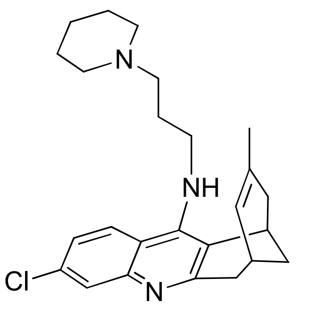

7

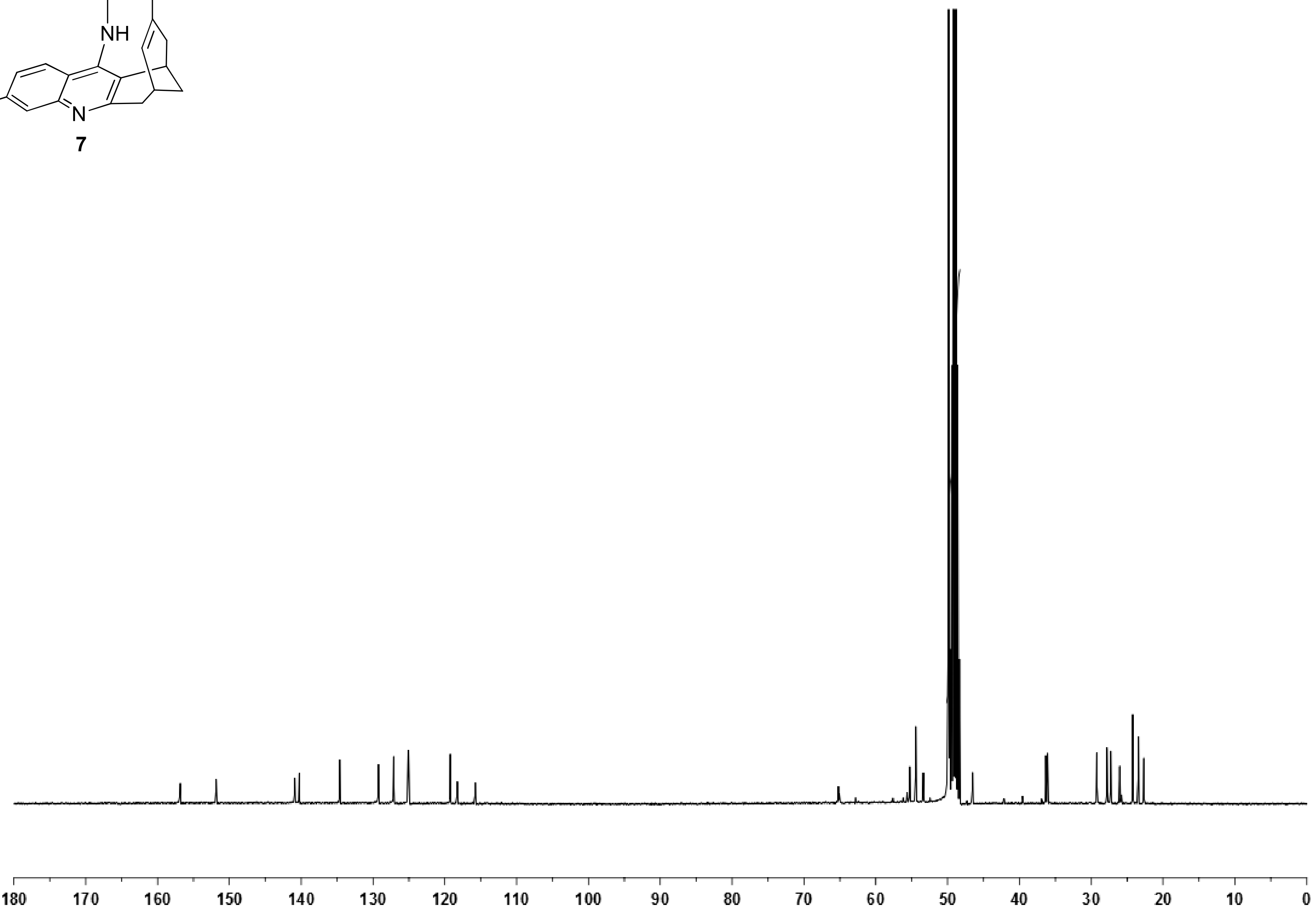

^{13}C NMR (100.6 MHz, CD_3OD)

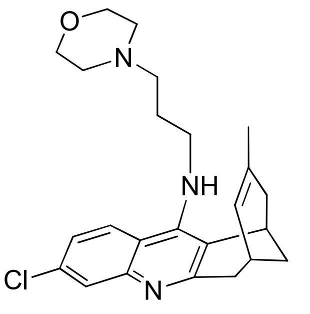

8

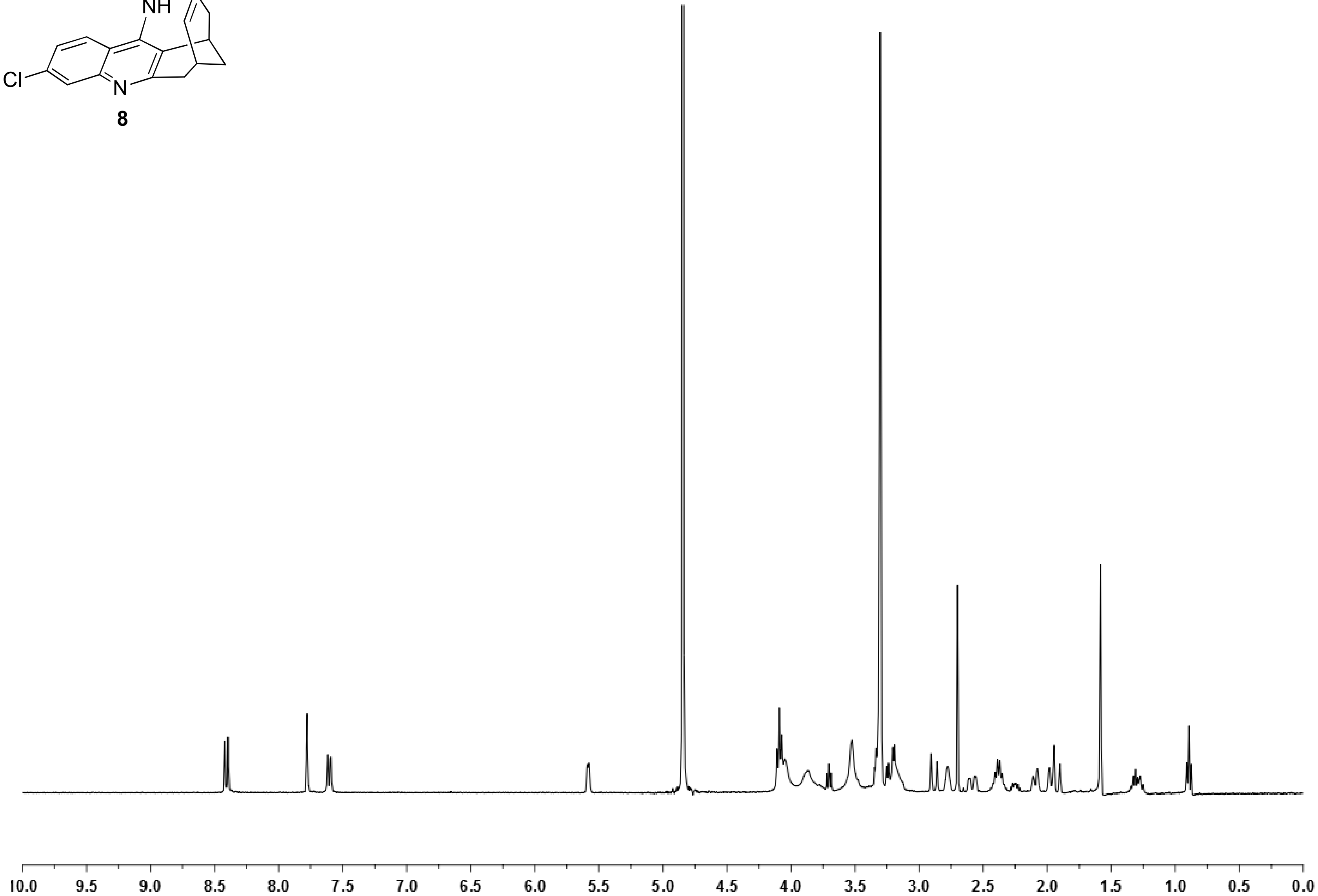

^{1}H NMR (400 MHz, CD_3OD)

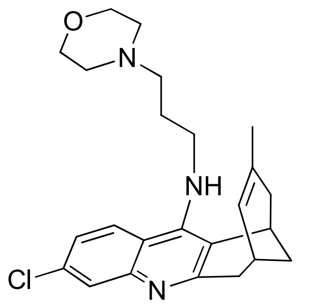

8

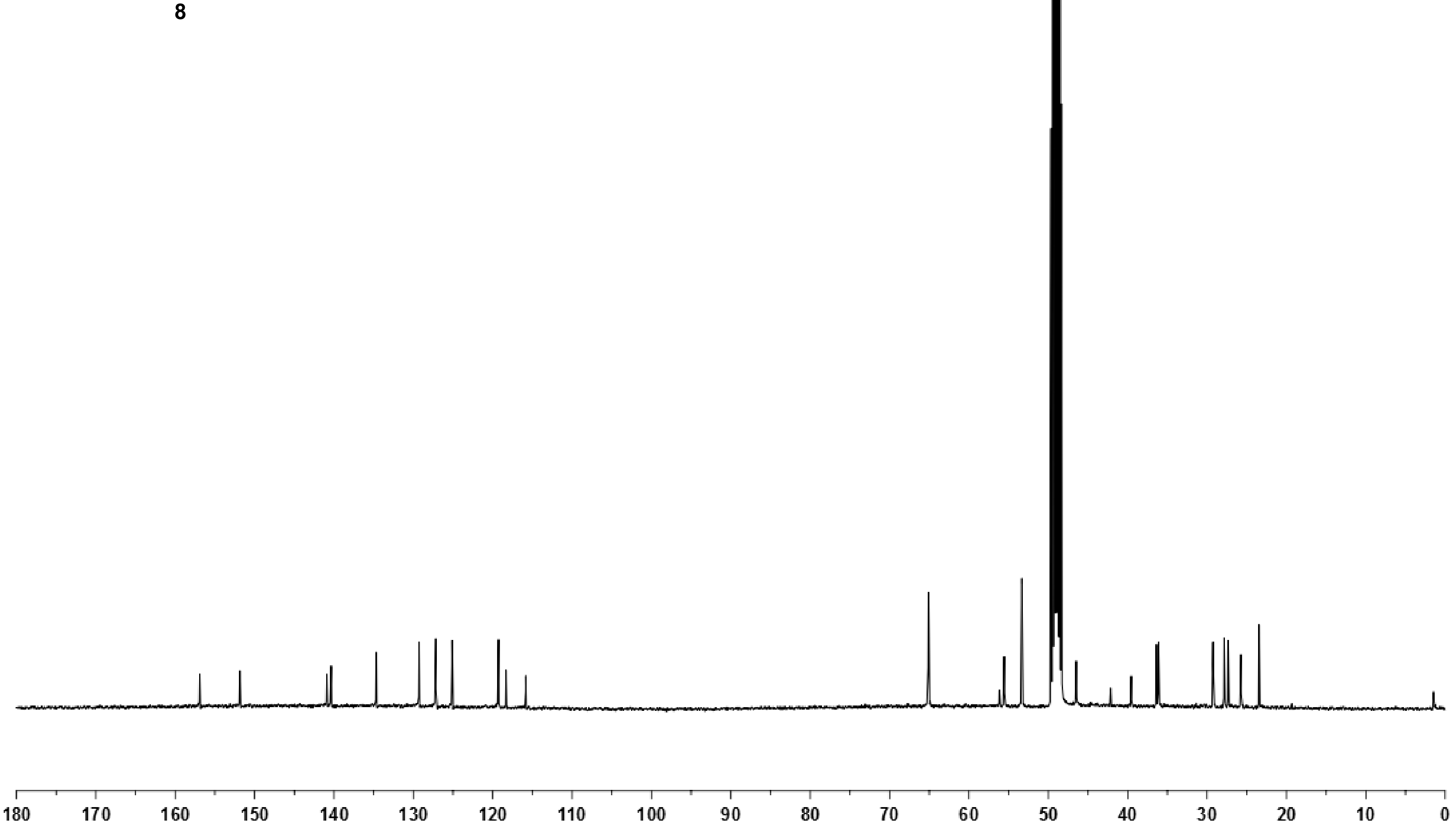

^{13}C NMR (100.6 MHz, CD_3OD)

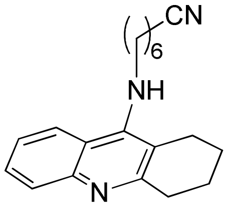

11c

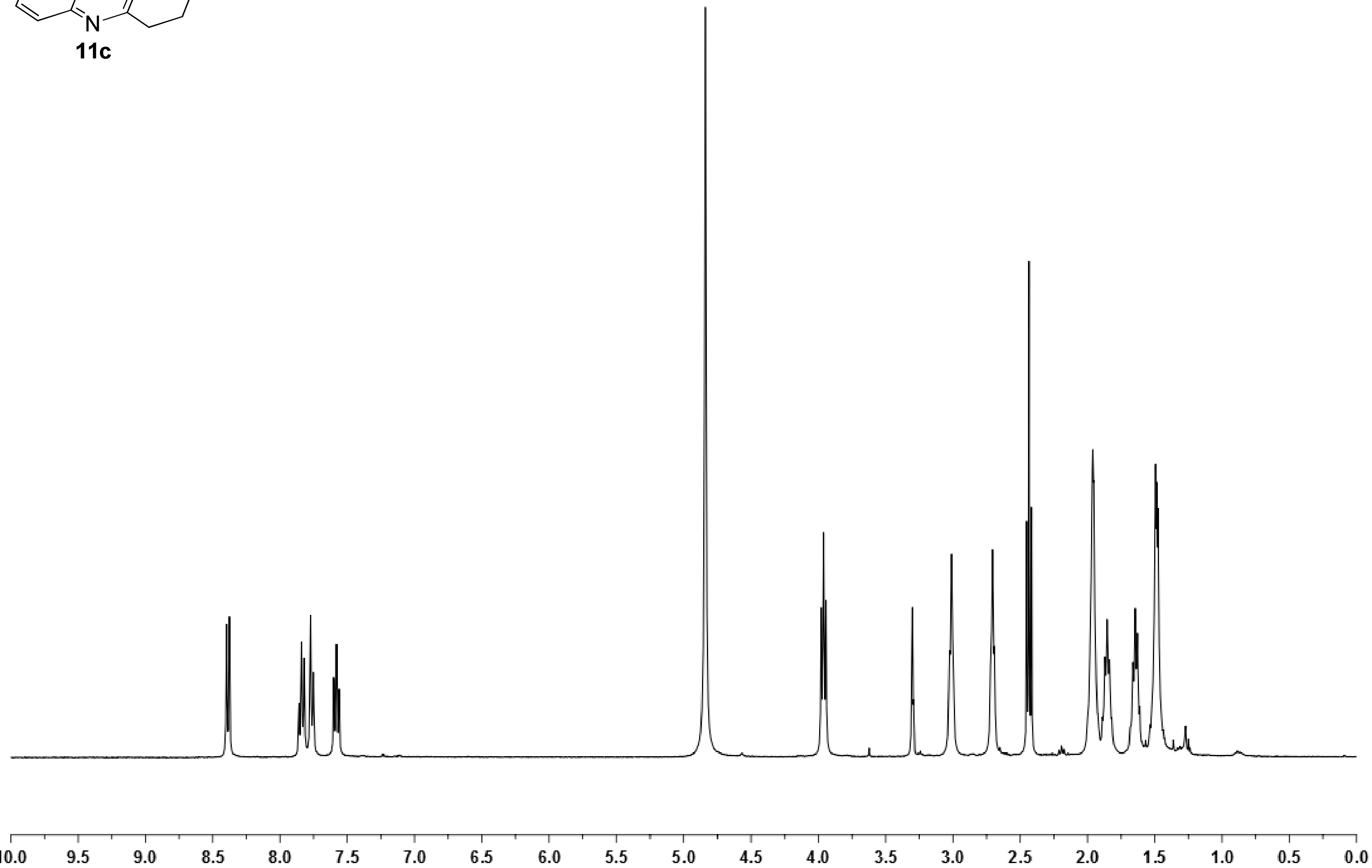

^{1}H NMR (400 MHz, CD_3OD)

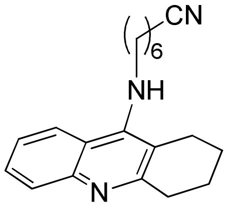

11c

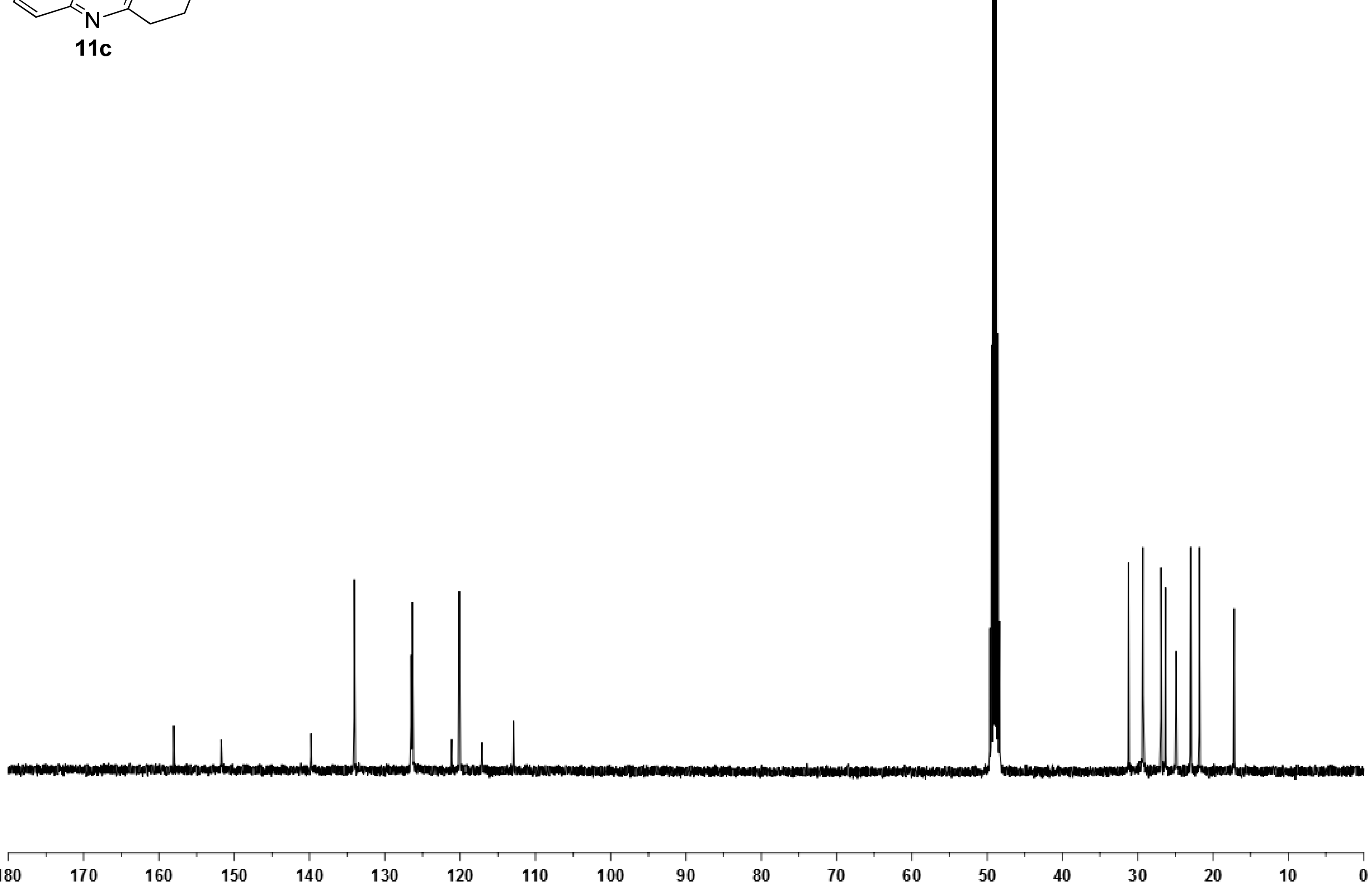

^{13}C NMR (100.6 MHz, CD_3OD)

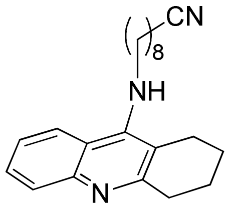

11e

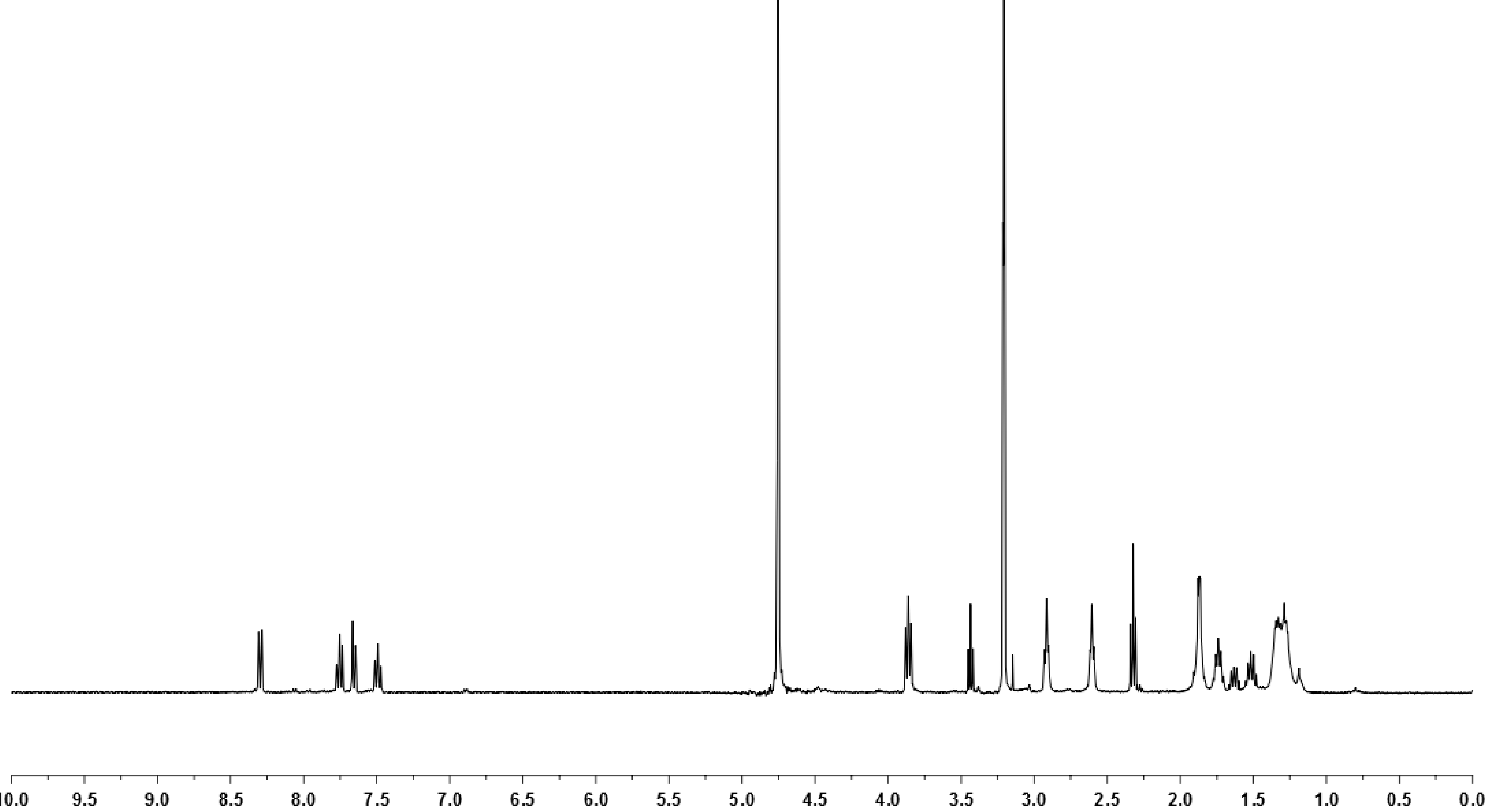

^{1}H NMR (400 MHz, CD_3OD)

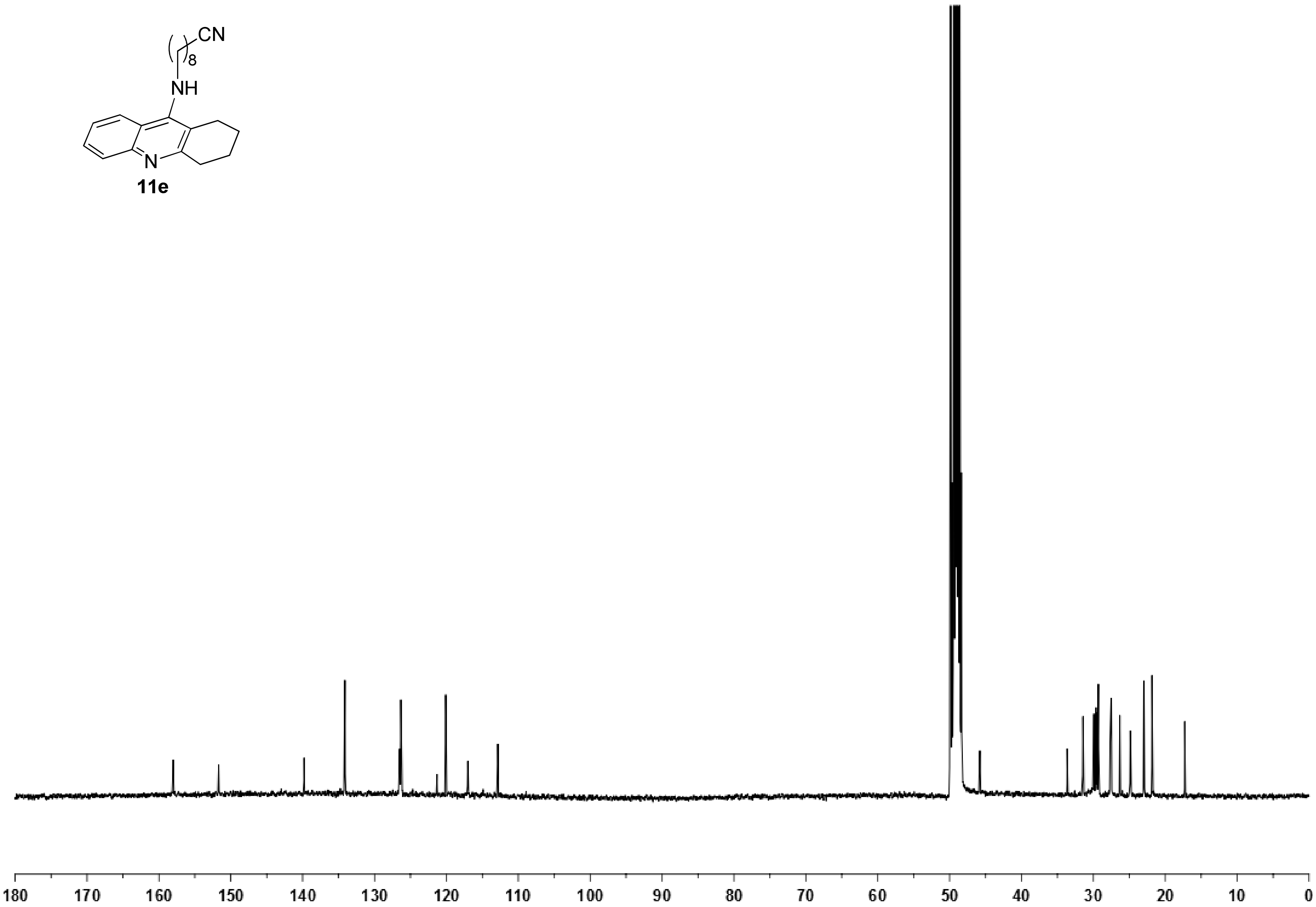

^{13}C NMR (100.6 MHz, CD_3OD)

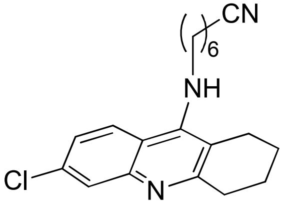

12c

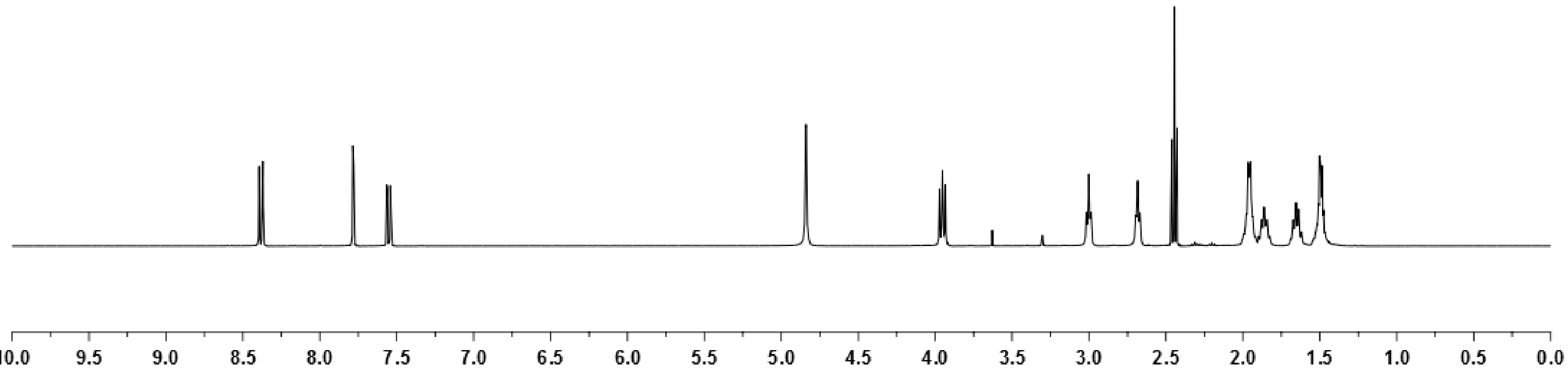

^{1}H NMR (400 MHz, CD_3OD)

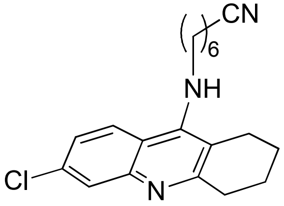

12c

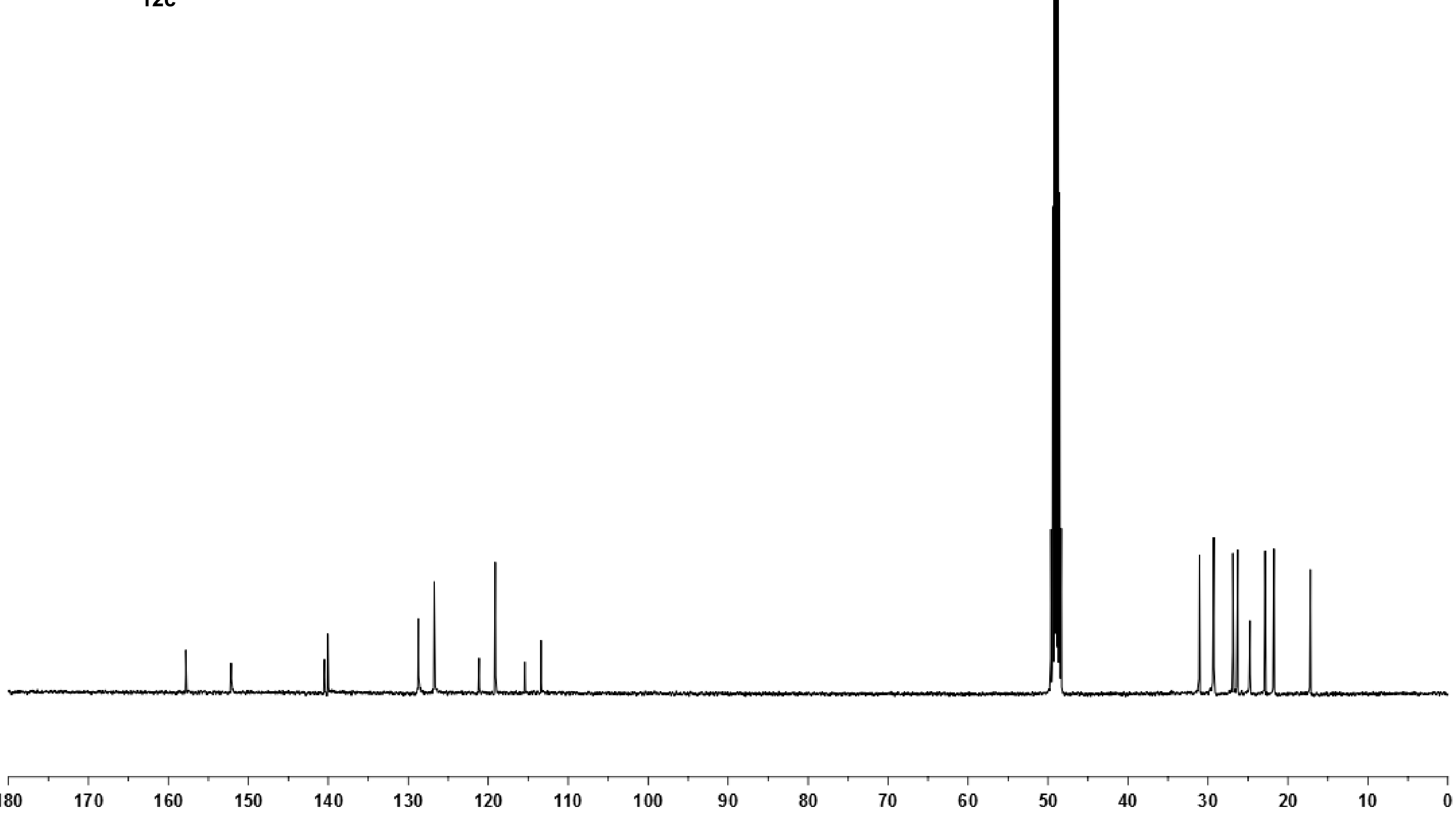

^{13}C NMR (100.6 MHz, CD_3OD)

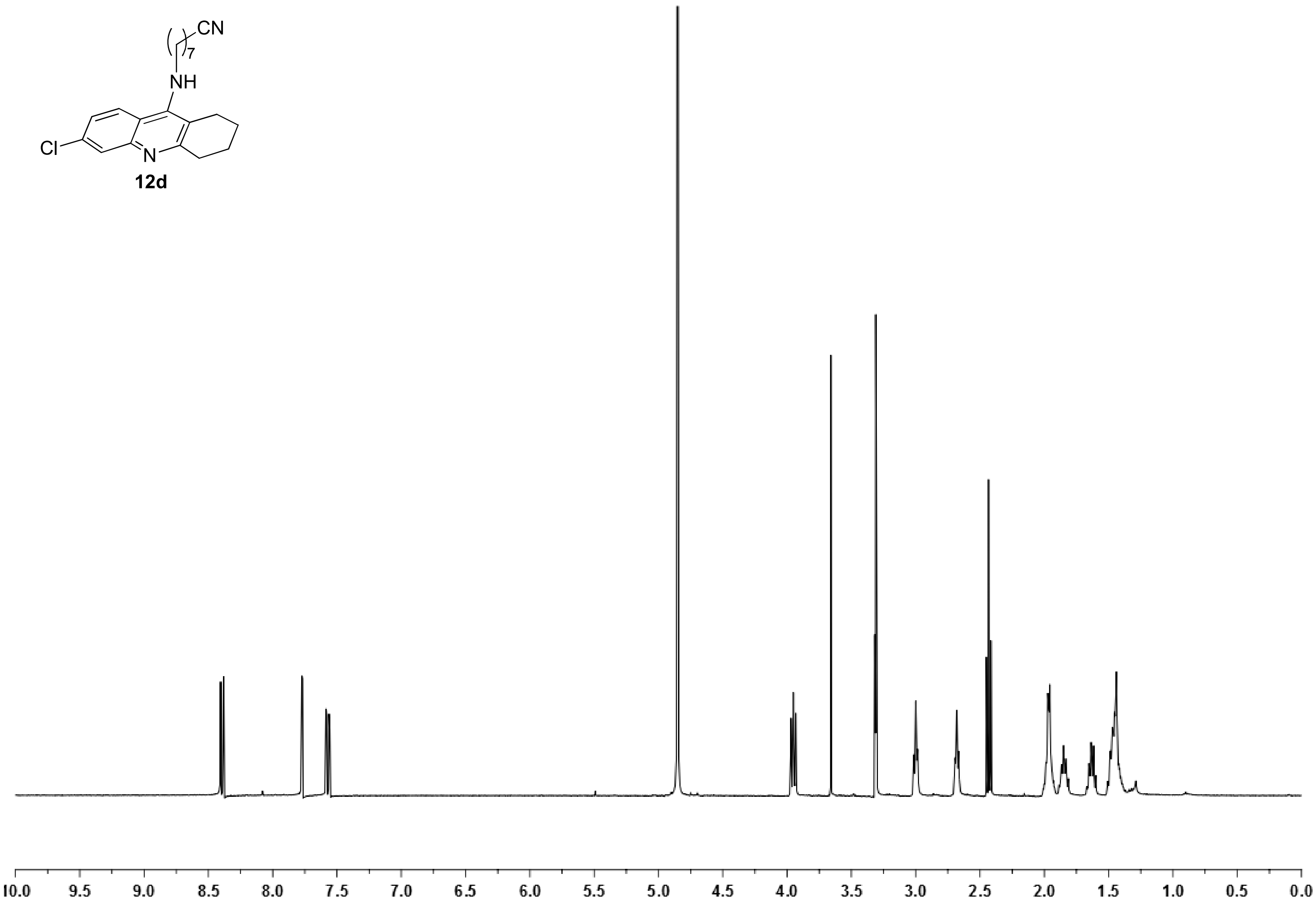

^1H NMR (400 MHz, CD_3OD)

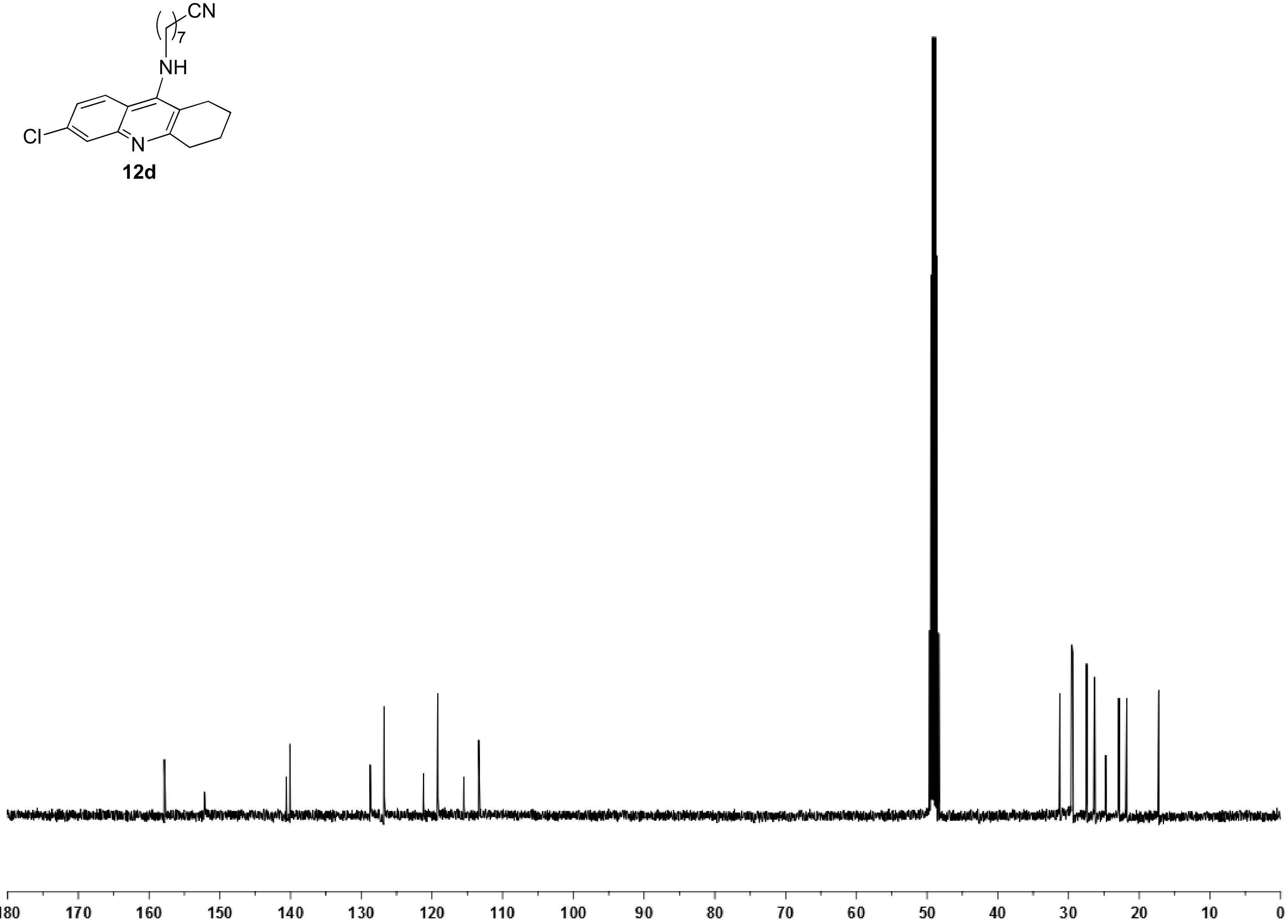

12d

^{13}C NMR (100.6 MHz, CD_3OD)

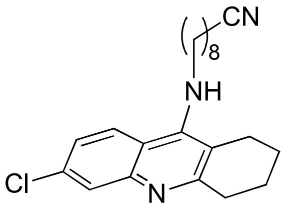

12e

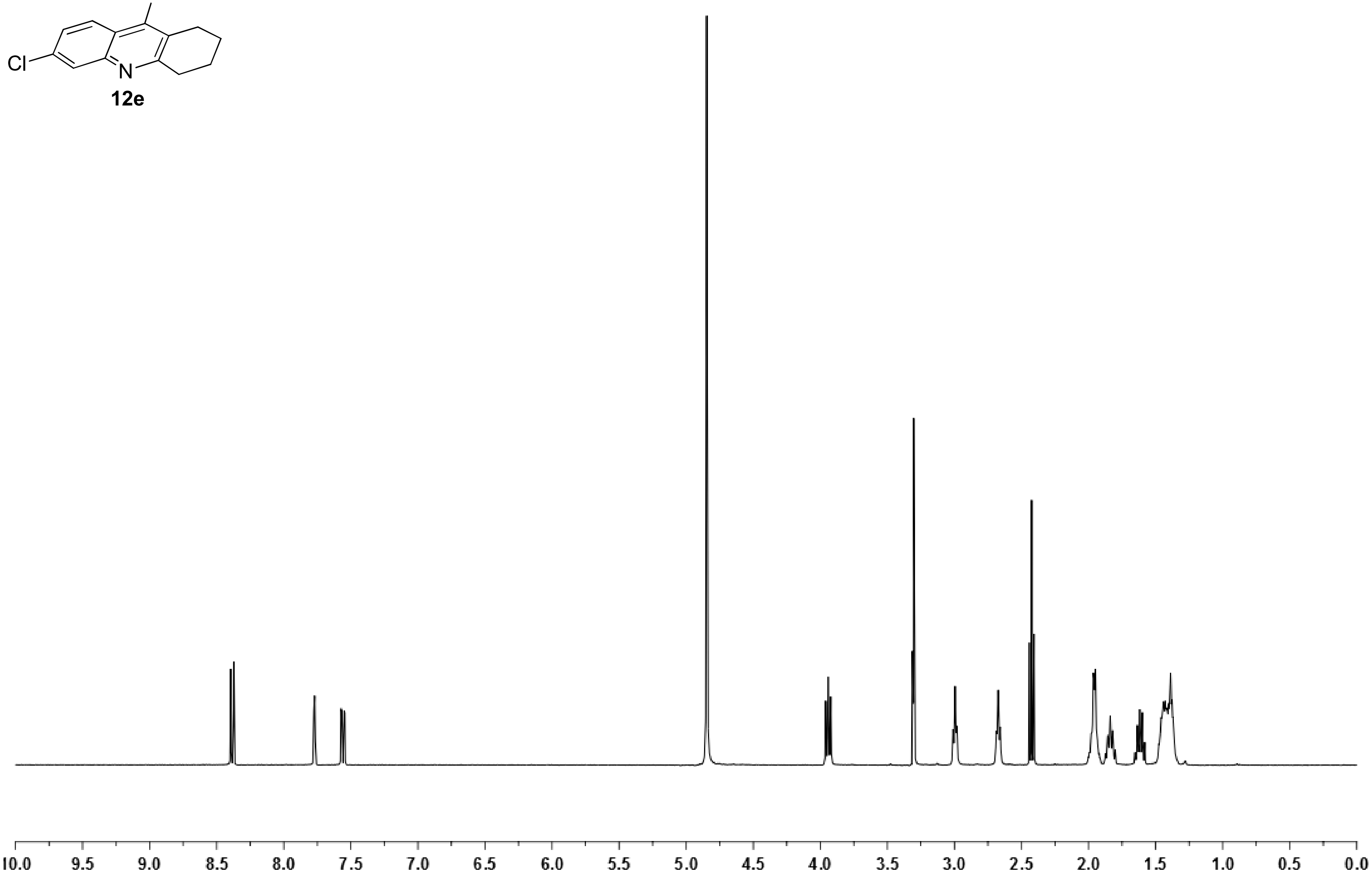

^{1}H NMR (400 MHz, CD_3OD)

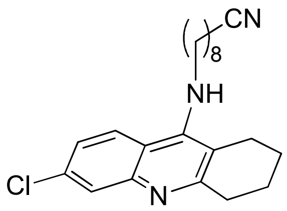

12e

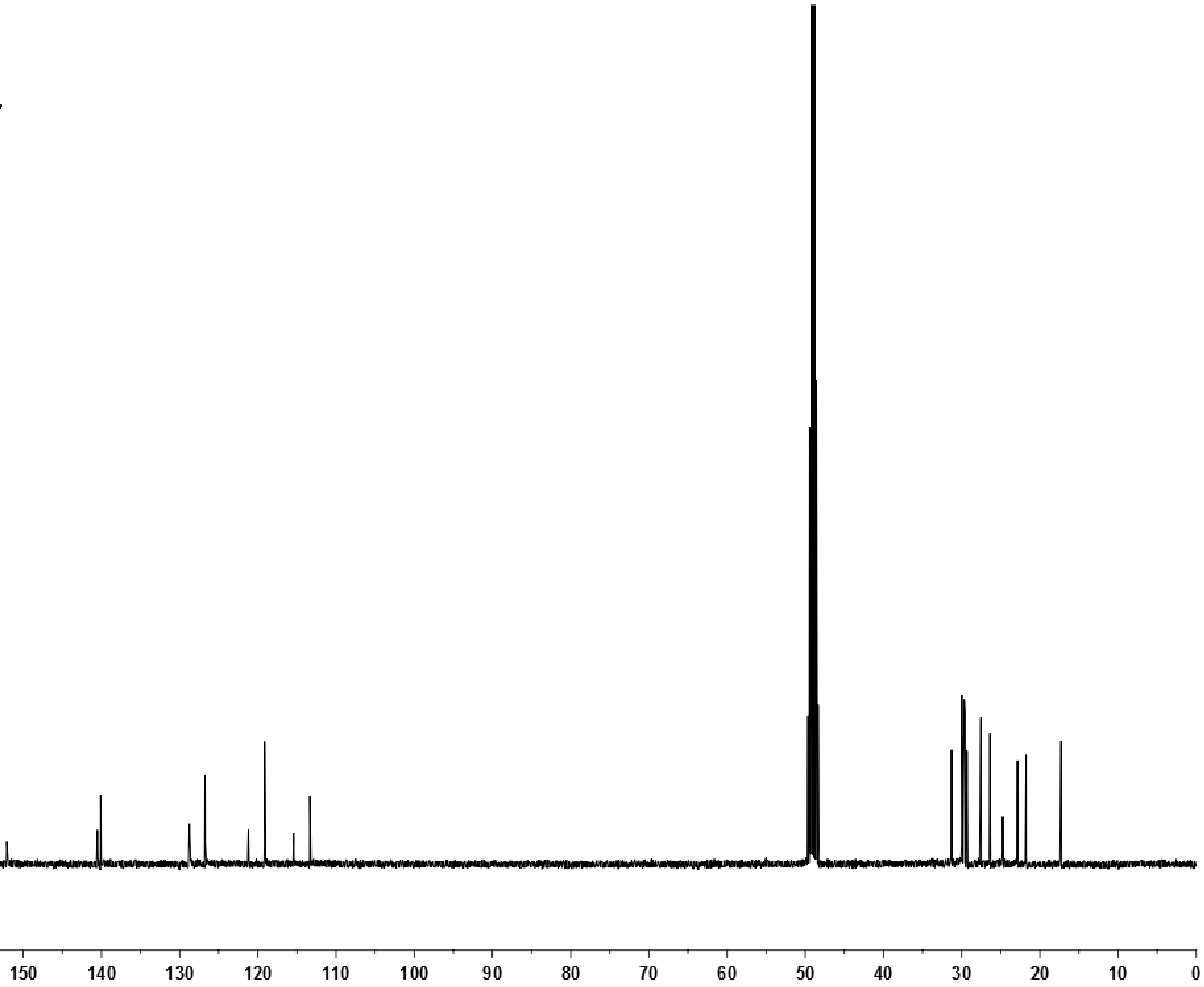

^{13}C NMR (100.6 MHz, CD_3OD)

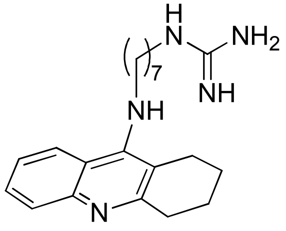

15c

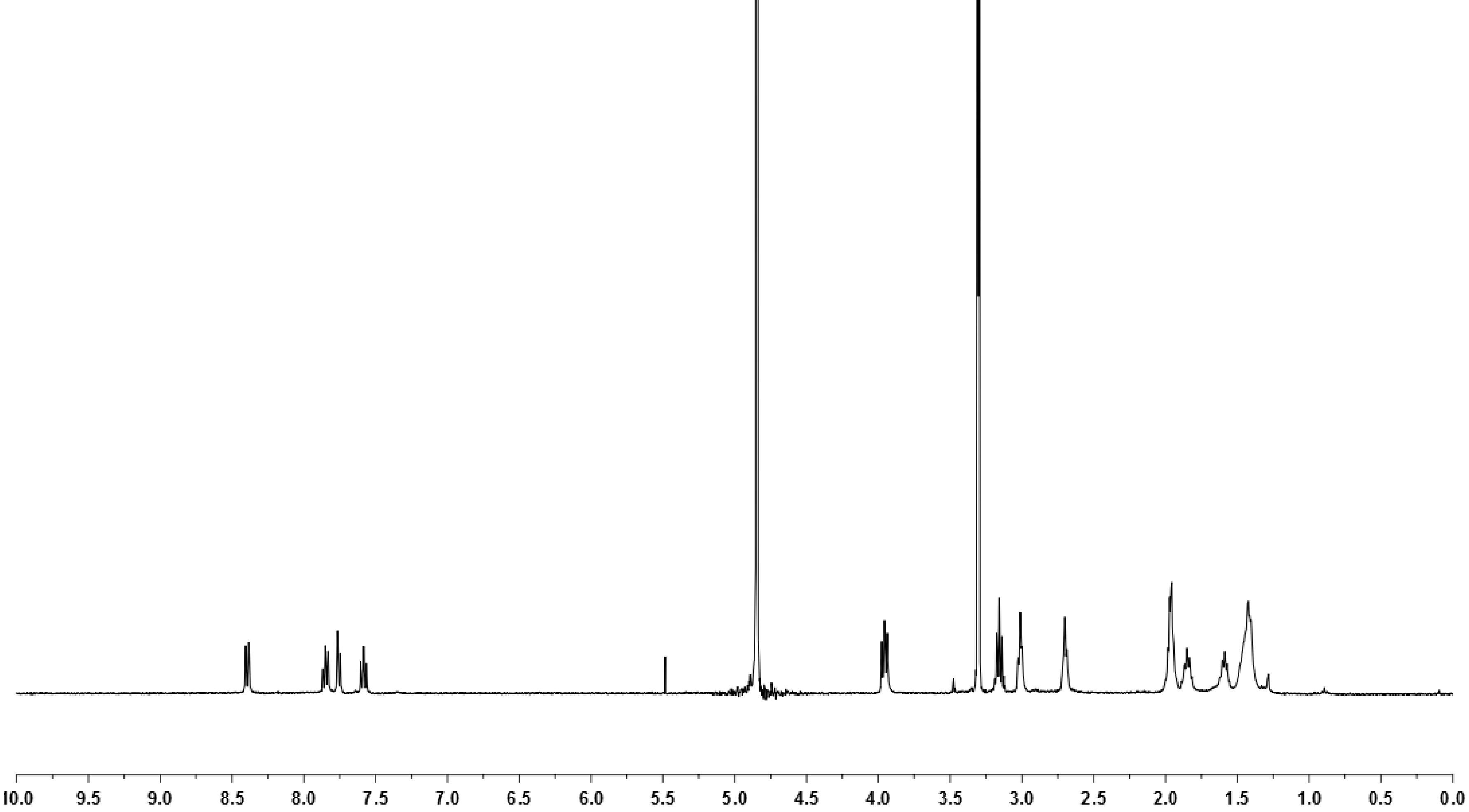

^{1}H NMR (400 MHz, CD_3OD)

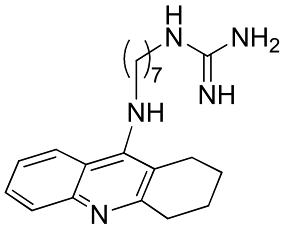

15c

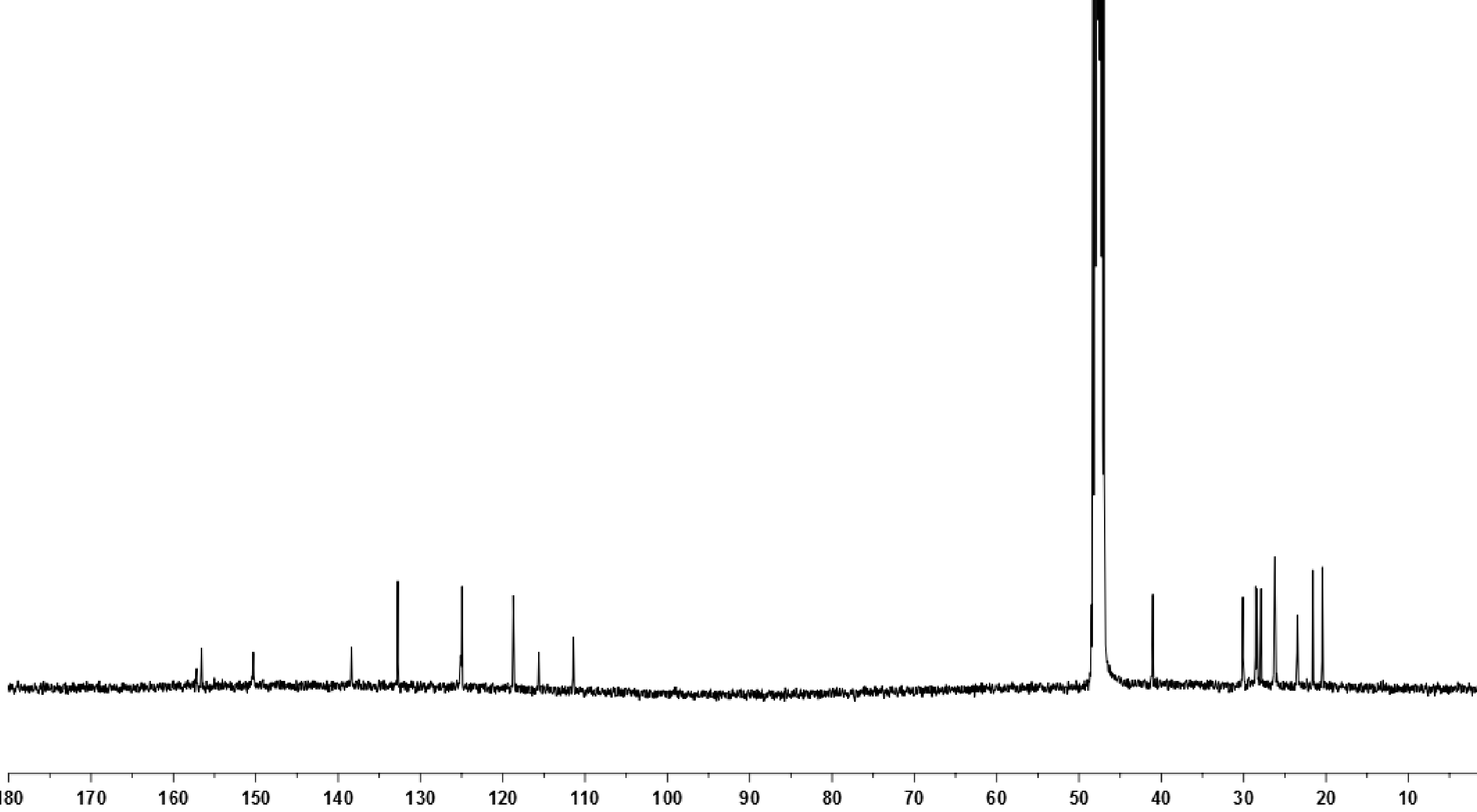

^{13}C NMR (100.6 MHz, CD_3OD)

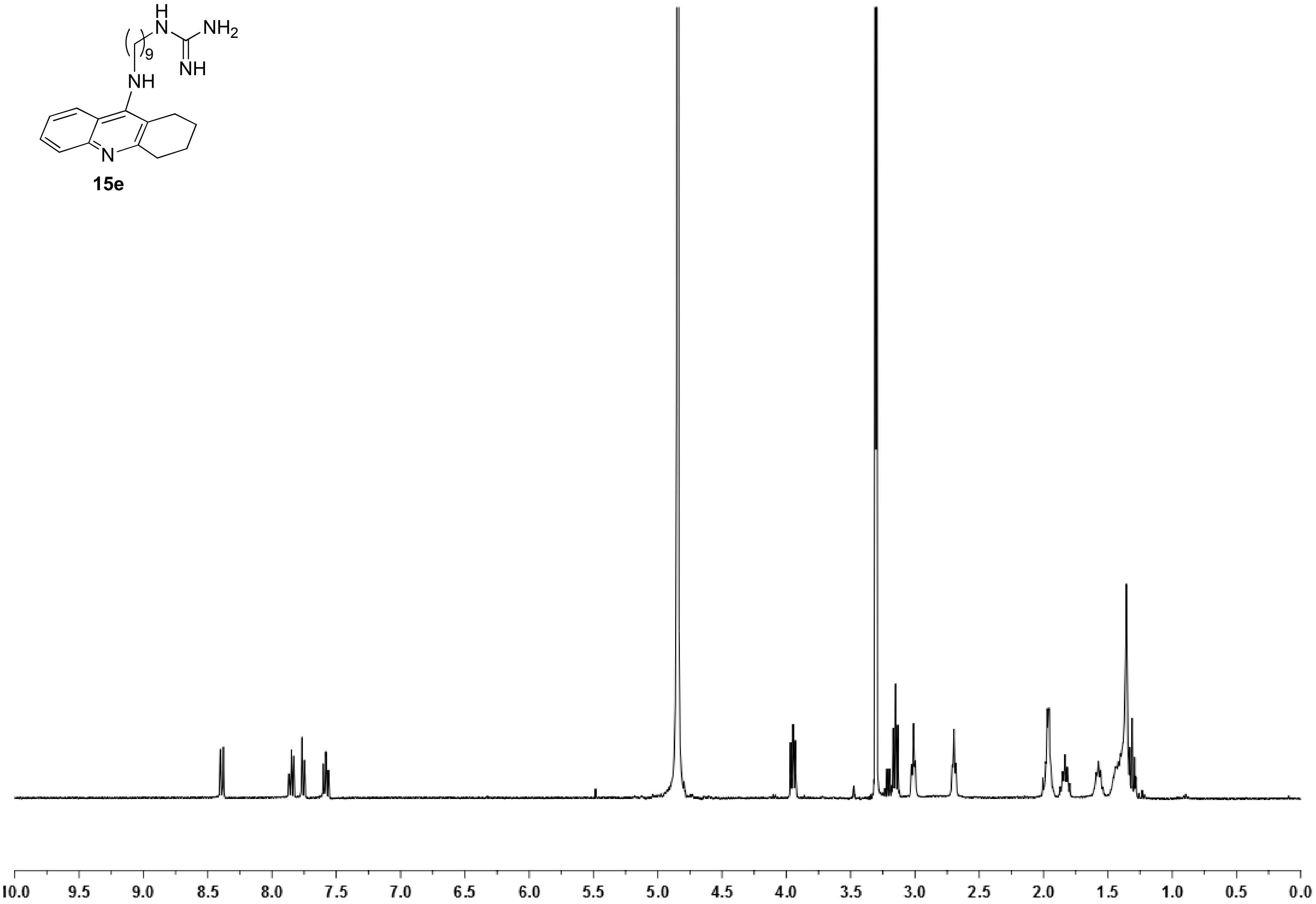

^{1}H NMR (400 MHz, CD_3OD)

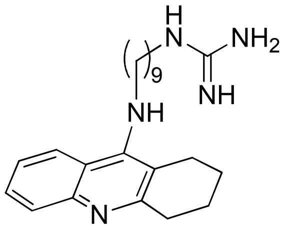

15e

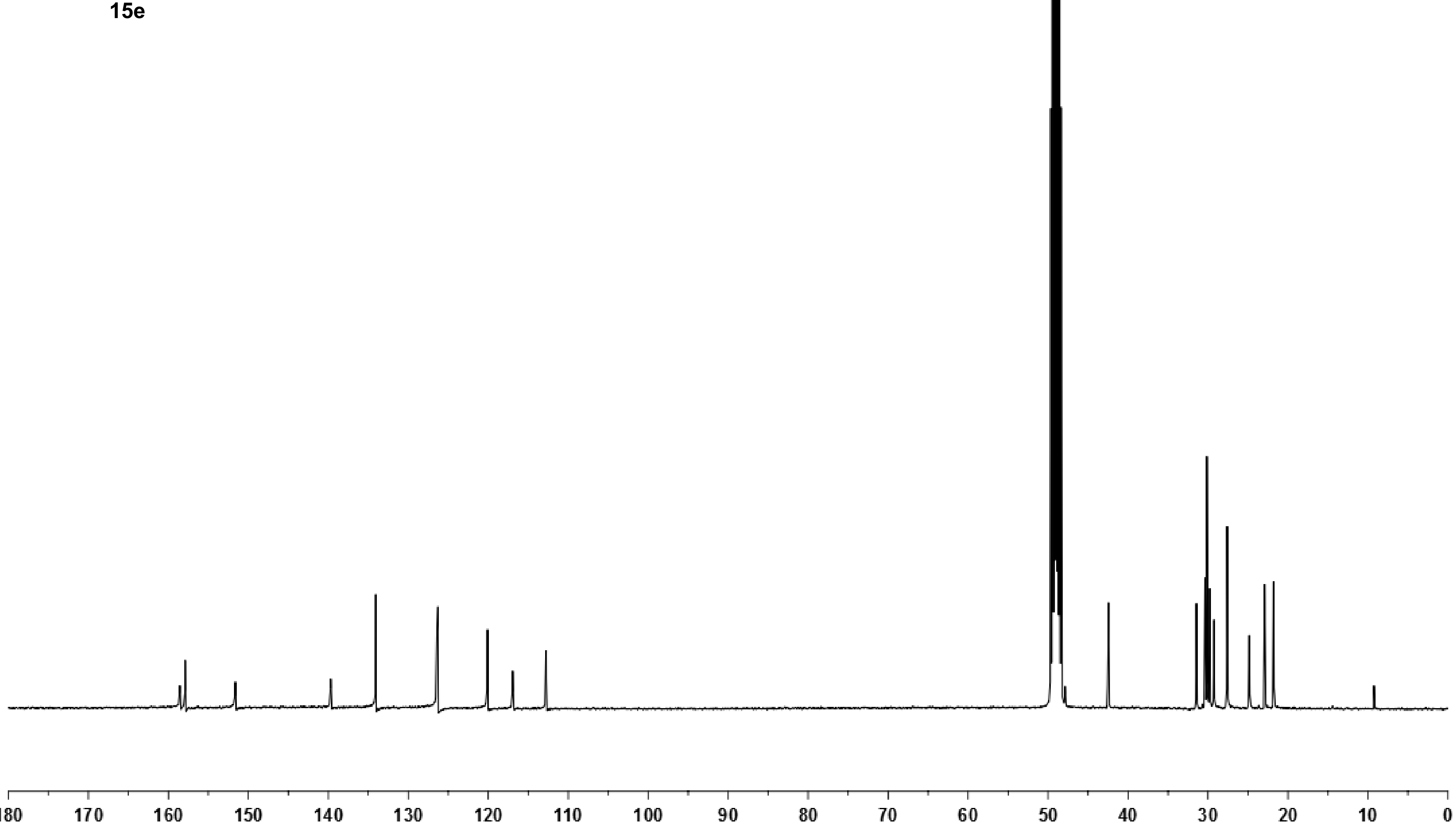

^{13}C NMR (100.6 MHz, CD_3OD)

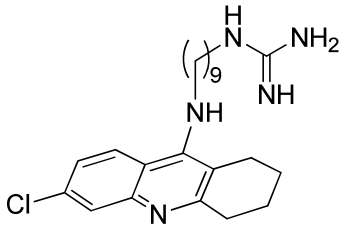

16e

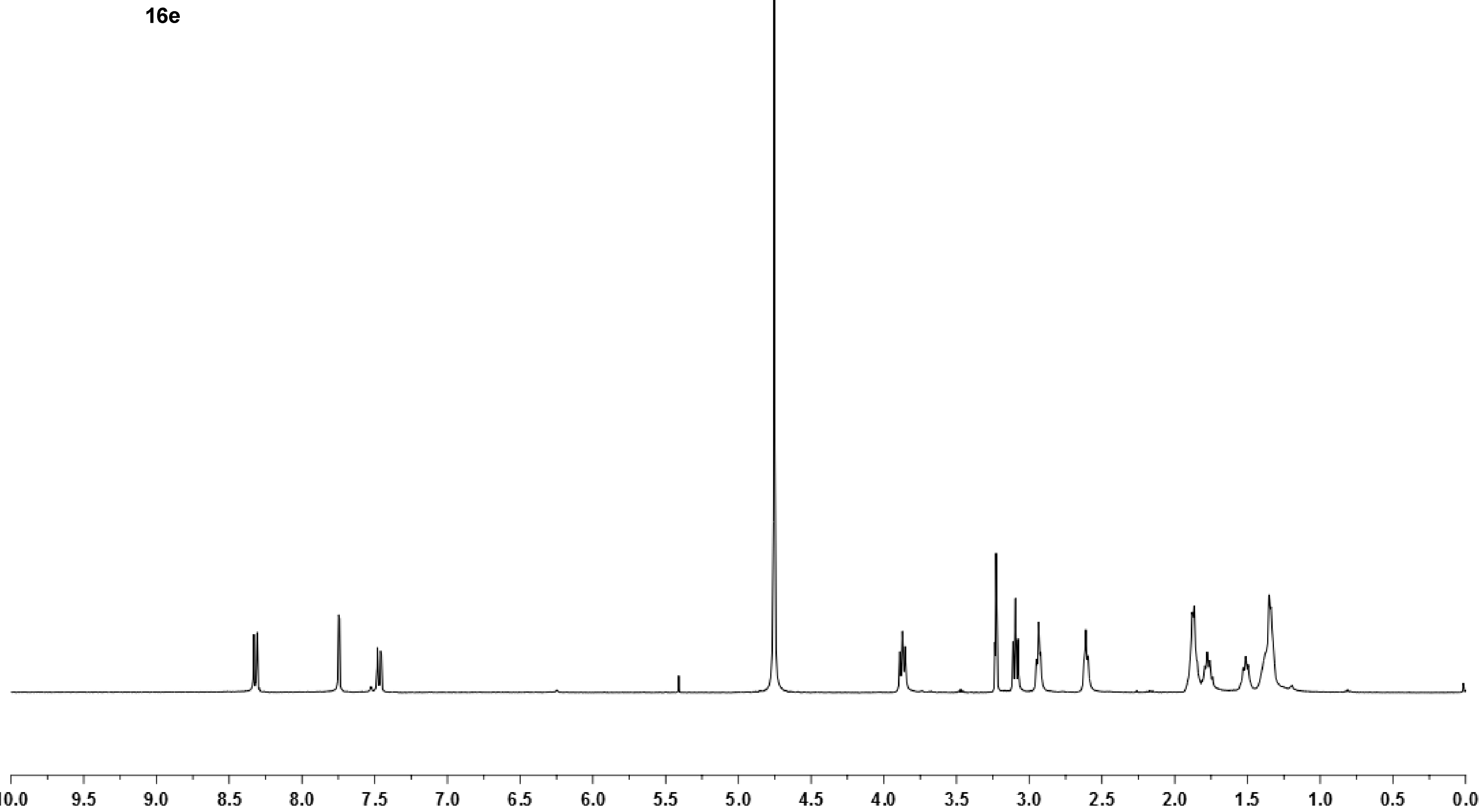

^{1}H NMR (400 MHz, CD_3OD)

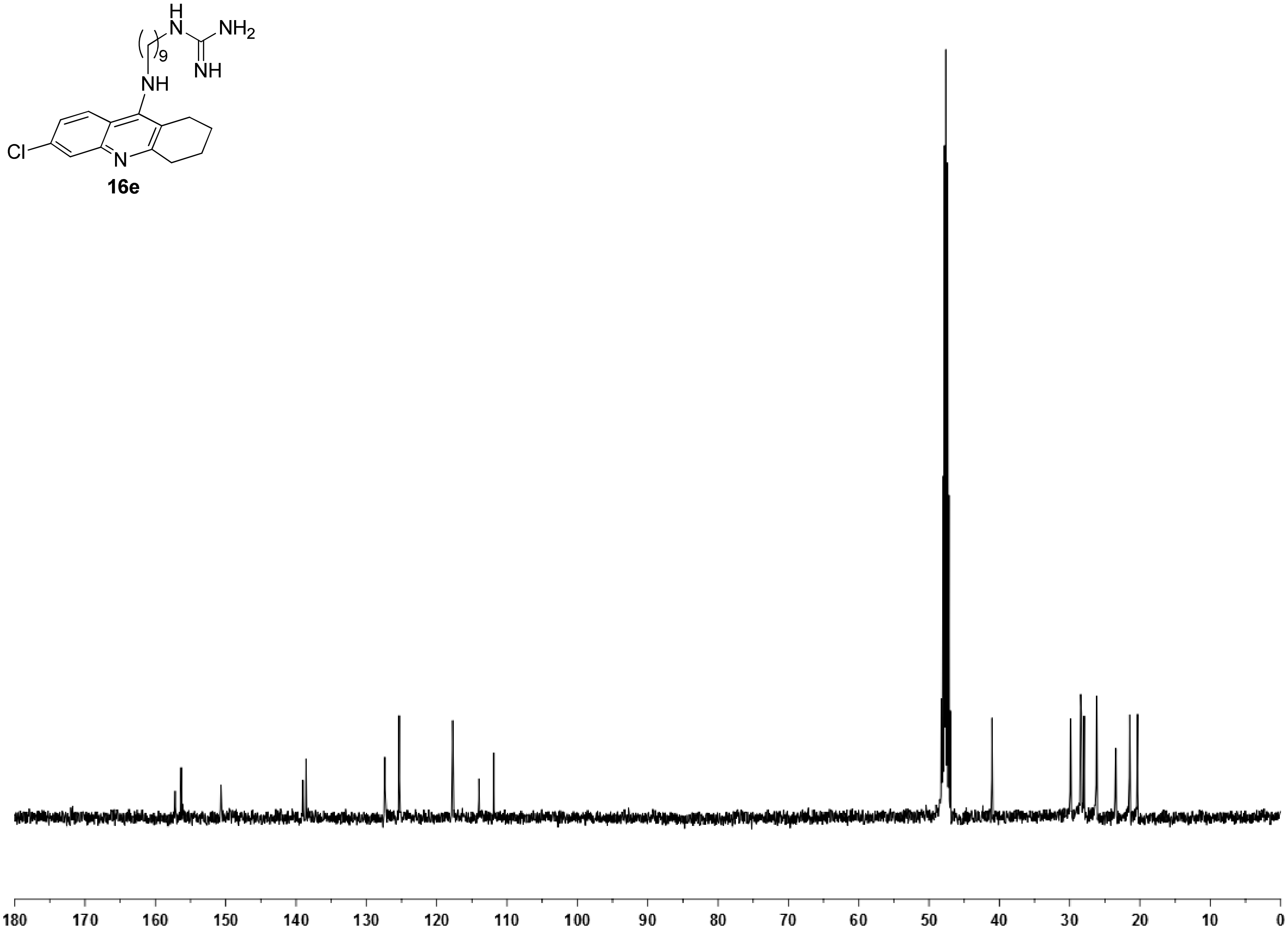

^{13}C NMR (100.6 MHz, CD_3OD)
